# The newly identified MEK1 tyrosine phosphorylation target MACC1 is druggable by approved MEK1 inhibitors to restrict colorectal cancer metastasis

**DOI:** 10.1038/s41388-021-01917-z

**Published:** 2021-07-10

**Authors:** Dennis Kobelt, Daniel Perez-Hernandez, Claudia Fleuter, Mathias Dahlmann, Fabian Zincke, Janice Smith, Rebekka Migotti, Oliver Popp, Susen Burock, Wolfgang Walther, Gunnar Dittmar, Philipp Mertins, Ulrike Stein

**Affiliations:** 1 Translational Oncology of Solid Tumors, Experimental and Clinical Research Center, Charité—Universitätsmedizin Berlin and Max-Delbrück-Center for Molecular Medicine in the Helmholtz Association, Berlin, Germany; 2 German Cancer Consortium (DKTK), Heidelberg, Germany; 3 Mass Spectrometry Core Unit, Max-Delbrück-Center for Molecular Medicine in the Helmholtz Association, Berlin, Germany; 4 Berlin Institute of Health (BIH), Berlin, Germany; 5 Charité Comprehensive Cancer Center, Charité—Universitätsmedizin Berlin, Berlin, Germany; 6 Proteome and Genome Research Laboratory, Luxembourg Institute of Health, Strassen, Luxembourg

**Keywords:** Oncogenes, Cell migration, Phosphorylation, Biomarkers

## Abstract

Cancer metastasis causes >90% of cancer deaths and remains a major treatment challenge. Here we deciphered the impact of tyrosine phosphorylation of MACC1, a causative driver for cancer metastasis, for cancer cell signaling and novel interventions to restrict cancer metastasis. We identified MACC1 as new MEK1 substrate. MEK1 directly phosphorylates MACC1, leading to accelerated and increased ERK1 activation. Mutating in silico predicted hierarchical MACC1 tyrosine phosphorylation sites abrogates MACC1-induced migration, invasion, and MET expression, a transcriptional MACC1 target. Targeting MEK1 by RNAi or clinically applicable MEK1 inhibitors AZD6244 and GSK1120212 reduces MACC1 tyrosine phosphorylation and restricts MACC1-induced metastasis formation in mice. Although MEK1 levels, contrary to MACC1, are not of prognostic relevance for CRC patients, MEK1 expression was found indispensable for MACC1-induced metastasis. This study identifies MACC1 as new MEK1 substrate for tyrosine phosphorylation decisively impacting cell motility, tumor growth, and metastasis. Thus, MAP kinase signaling is not linear leading to ERK activation, but branches at the level of MEK1. This fundamental finding opens new therapeutic options for targeting the MEK1/MACC1 axis as novel vulnerability in patients at high risk for metastasis. This might be extended from CRC to further solid tumor entities.

## Introduction

Colorectal cancer (CRC) metastasis is directly linked to patient survival and accounts for 90% of patient deaths. It critically limits successful therapy, representing the most frequent cause of treatment failure [1–3]. Biomarkers for metastasis prediction, for identification of high-risk patients at early stages, and as targets for therapeutic interventions are therefore of upmost importance.

We previously identified the gene Metastasis Associated in Colon Cancer 1 (MACC1) in human CRC [4]. MACC1 induces cell proliferation, dissemination, migration, and invasion in cell culture, as well as tumor progression and metastasis in mouse models [4–7]. MACC1 transcripts in CRC patient’s tumor tissue and blood predict metastasis formation and patient survival, thereby also allowing the early identification of high-risk patients [4, 6, 8–14,]. Novel detection platforms for ultra-sensitive detection of MACC1 transcripts in liquid biopsies and tissue are currently in active development [15]. MACC1 has been confirmed as prognostic and predictive biomarker in a variety of solid cancers like CRC, gastric, esophageal, pancreatic, hepatocellular/biliary, lung, ovarian, breast, renal, bladder, nasopharyngeal cancer, glioblastoma, and osteosarcoma [16–24]. MACC1 expression levels correlate with tumor formation, progression, metastasis, and patient survival. This affirms MACC1 as a decisive driver for tumor growth and metastasis, but also as a therapeutic target to restrict cancer progression and metastasis (reviewed in ref. [25]). Proof-of-concept was provided by transcriptional and post-transcriptional downregulation of MACC1 for hepatocellular, ovarian, renal, and CRC [16, 24, 26, 27]. However, specific inhibitors targeting MACC1 post-translational protein modifications, to restrict tumor growth and metastasis, are not identified so far.

One of the most highly dynamic and actively researched type of post-translational modifications is protein phosphorylation [28]. The primary structure of MACC1 contains several sites of predicted tyrosine phosphorylation. Although tyrosine phosphorylation occurs only in 0.05% of the phospho-proteome of eukaryotic cells, it is essential for signal transduction, proliferation, cell-cycle progression, metabolism, differentiation, and development [29]. Defective tyrosine phosphorylation underlies many human diseases, particularly cancer [30, 31]. Their importance for MACC1 is not yet discovered.

Here we identified for the first time the metastasis inducer MACC1 as novel target for MEK1. MEK1-driven tyrosine phosphorylation of MACC1 is necessary for the motility and metastasis-inducing function of MACC. Furthermore, we demonstrate that clinically relevant MEK1 inhibitors restrict MACC1-induced cellular motility and metastasis.

## Results

### MEK1 inhibitors restrict MACC1-induced cell motility in vitro

**Fig. 1:**
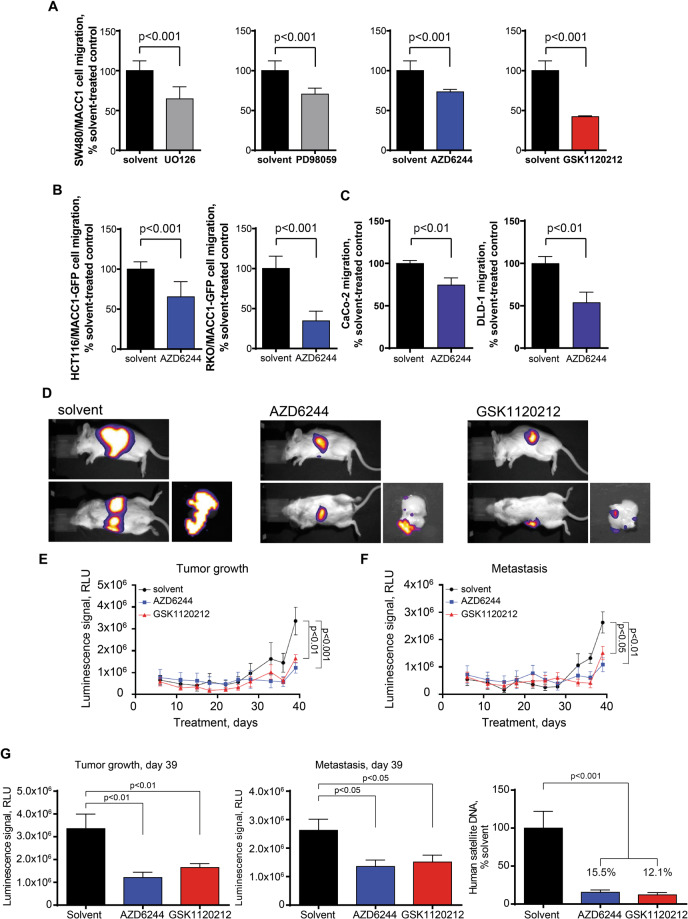
Targeting MEK1 restricts MACC1-induced cellular motility, tumor growth, and metastasis in mice. All cell lines show increased migration when MACC1 is overexpressed. **A** MEK1 inhibitors restrict MACC1-induced cell migration in vitro. SW480/luc-MACC1 cells were treated with DMSO, 20-µM UO126, 50-µM PD98059, 10-µM AZD6244, or 10-µM GSK1120212. Cell migration was significantly inhibited by all MEK1 inhibitors compared to solvent-treated controls (all *p* < 0.001). Data are presented as mean with SD. The finding from SW480/MACC1 cells was further validated in cell lines with forced MACC1 overexpression (HCT116/MACC1, RKO/MACC1) **(B)** and cell lines with high endogenous MACC1 overexpression (CaCo-2, DLD-1) **(C)**. CRC cells were treated with 10-µM AZD6244. For all cell lines, MEK1 inhibition results in reduced MACC1-induced migration. Data are presented as mean with SD. To monitor restriction of tumor growth and metastasis formation induced by MACC1 SW480/luc-MACC1 cells were injected into the spleen of SCID beige mice. Treatments started at the day of transplantation and were continued until the animals were sacrificed. MEK1 inhibitors AZD6244 and GSK1120212 restrict MACC1-induced tumor growth and liver metastasis formation in mice. Mice were treated daily with either solvent, 50-mg/kg AZD6244 twice a day, or 2-mg/kg GSK1120212 once a day (*n* = 6 mice/group). In vivo bioluminescence imaging was documented twice per week. At the endpoint, ex vivo bioluminescence imaging was performed with the isolated organs of all animals. **D** Bioluminescence signals of solvent- (left), AZD6244- (middle), or GSK1120212-treated (right) SW480/Luc and SW480/Luc-MACC1 xenografted mice, shown for the lateral (upper panel) and ventral (lower panel) view. In addition, isolated livers are shown. Reduction of signals was observed for MEK1 inhibitor-treated animals, as exemplified for one representative mouse. Treatments started at the day of transplantation and were continued until the animals were sacrificed. **E** Tumor growth is significantly reduced by both MEK1 inhibitors over time (solvent vs. AZD6244 *p* < 0.001; solvent vs. GSK1120212 *p* < 0.01, two-way ANOVA). **F** Metastasis is significantly reduced by both MEK1 inhibitors over time (solvent vs. AZD6244 *p* < 0.01; solvent vs. GSK1120212 *p* < 0.05, two-way ANOVA). **G** At the experimental endpoint tumor growth (solvent vs. AZD6244 *p* < 0.01; solvent vs. GSK1120212 *p* < 0.01, one-way ANOVA) and metastasis (solvent vs. AZD6244 *p* < 0.05; solvent vs. GSK1120212 *p* < 0.05, one-way ANOVA) were significantly delayed. Quantification of the amount of human cells in the livers of all SW480/luc-MACC1-transplanted mice was performed by amplification of human satellite DNA. Human satellite DNA was significantly reduced in MEK1 inhibitor-treated animals illustrating their restricted metastatic potential (both *p* < 0.001, one-way ANOVA). Data are presented as mean with SEM.

In our seminal discovery of MACC1, we identified this gene as prognostic biomarker for CRC. MEK1 inhibitors UO126 and PD98059 restricted MACC1-induced cell scattering, indicating that MAPK signaling is important for MACC1 function [4]. Here, we tested these MEK1 inhibitors to modulate MACC1-induced cell migration in SW480/luc-MACC1 cells, including FDA-approved GSK1120212 (JTP74057, trametinib) and AZD6244 (ARRY142886, selumetinib), already used in clinical trials. All MEK1 inhibitors reduced migration of SW480/luc-MACC1 cells, expressing firefly luciferase and MACC1: UO126 to 64%, PD98059 to 70%, AZD6244 to 73%, GSK1120212 to 42% (all *p* < 0.001; Fig. 1A).

These results were verified in additional cancer cell lines, HCT116/MACC1-GFP, RKO/MACC1-GFP, CaCo-2 and DLD-1 (Fig. 1B-C): AZD6244 treatment reduced cell migration in HCT116/MACC1-GFP to 65% (*p* < 0.001), in RKO/MACC1-GFP to 34% (*p* = 0.002), in CaCo-2 to 75% (p = 0.005) and in DLD-1 to 54% (p = 0.008). These data confirm the functional importance of MAPK signaling and of the key player MEK1 for MACC1-induced cell phenotypes.

### MEK1 inhibitors restrict MACC1-induced tumor growth and metastasis in mice

We investigated clinically applicable MEK1 inhibitors on MACC1-induced tumor growth and metastasis in xenografted mice over time by in vivo bioluminescence imaging. SCID bg/bg mice intrasplenically implanted with SW480/luc-MACC1 cells were orally treated with solvent (daily), 50-mg/kg AZD6244 twice daily or 2-mg/kg GSK1120212 daily (Fig. 1D–G) [32, 33].

Starting on day 28, strongly increased luminescence signals were detected with significant differences between solvent- and MEK1 inhibitor-treated animals (Fig. 1E, F). At days 36 and 39, tumor growth and metastasis were delayed in MEK1 inhibitor-treated animals (day 39; controls: mean lateral signal 3.4 × 10^6^, CI 1.9 × 10^6^–4.8 × 10^6^, AZD6244: mean lateral signal 1.2 × 10^6^, CI 0.69 × 10^6^–1.7 × 10^6^, *p* < 0.01, GSK1120212: mean lateral signal 1.6 × 10^6^, CI 1.3 × 10^6^–2.0 × 10^6^, *p* < 0.01; controls: mean ventral signal 2.6 × 10^6^, CI 1.7 × 10^6^–3.5 × 10^6^, AZD6244: mean ventral signal 1.4 × 10^6^, CI 0.8 × 10^6^–1.9 × 10^6^, *p* < 0.05, GSK1120212: mean ventral signal 1.5 × 10^6^, CI 0.97 × 10^6^–2.1 × 10^6^, *p* < 0.05, Fig. 1E–G). Reduced metastasis in MEK1 inhibitor-treated animals was confirmed visually, by ex vivo imaging of the liver, and by human microsatellite DNA (controls: mean = 100% CI 53.9–146.1%, AZD6244: mean = 15.5% CI 9–22%, GSK1120212: mean = 12.1% CI 6–18.2%; both *p* < 0.001) (Fig. 1D, G).

These results were confirmed by an additional mouse experiment using RKO/MACC1 cells intrasplenically xenotransplanted in mice. Overexpression of MACC1 induced metastasis formation, whereas MEK1 downregulation by shRNA or inhibition by the small molecule inhibitor AZD6244 reduced MACC1-induced metastasis formation (Fig. S1A–C).

Taken together, the metastatic abilities of CRC cells provoked by MACC1-wt transfection are dependent on MEK1. This dependence can be capitalized for MEK1 inhibitor-based restriction of MACC1-induced metastasis.

### MEK1 binds and phosphorylates MACC1

**Fig. 2:**
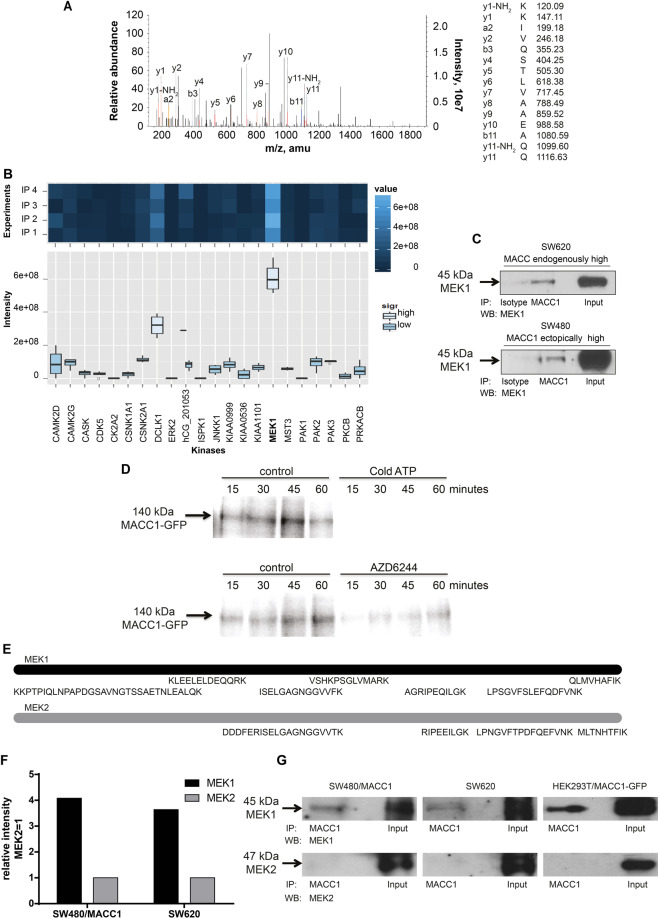
Identification of the dual specificity protein kinase MEK1 (MAP2K1) binding to MACC1 for tyrosine phosphorylation of MACC1. **A** Tandem MS-spectrum for a peptide derived from MACC1. The sequence of this peptide was verified by the presence of complementary b-ions (NH2 terminus-derived fragment ions) and y-ions (COOH terminus-derived fragment ions). **B** Identification of protein kinases interacting with MACC1 by quantitative proteomics. We performed two independent IPs of MACC1 from SW620 cells with high endogenous MACC1 expression employing two different MACC1 antibodies (four experiments), which were subjected to shot-gun MS. In total, 1203 proteins were identified, including 22 kinases (depicted). Highest signal intensities were measured for MEK1 (MAP2K1), a member of the dual specificity protein kinase family able to phosphorylate tyrosine and threonine residues. All other 21 kinases binding with lower intensities belong to the serine/threonine kinase family. **C** Validation of physical interaction of MACC1 and MEK1 was carried out in endogenously high MACC1-expressing SW620 cells (upper panel) and ectopically high MACC1-expressing SW480/MACC1-wt cells (lower panel). IP was performed for MACC1 and β-tubulin (control). Western blot was done using a MEK1 antibody. **D** Incubation of isolated MACC1-GFP from HEK293T cells with recombinant constitutively active MEK1 S218D S222D and γP32 ATP results in transfer of radioactive phosphate to MACC1 (each left panel). Non-labeled ATP completely out-competed labeled ATP (upper right panel). In the control reaction, cold ATP was omitted. The MEK1 inhibitor AZD6244 inhibited the transfer of radioactive phosphate to MACC1-GFP (lower right panel). In the control reaction, DMSO without inhibitor was added as solvent control. MACC1-GFP was isolated by nanotrap IP for GFP. One IP of 10-µg MACC1-GFP was incubated with 500-ng recombinant MEK1 S218D S222D at 37 °C. At indicated time points, 25% of the reaction was removed, supplemented with western blot loading buffer and boiled to stop the reaction. All samples were separated using polyacrylamide gel electrophoresis. The gels were dried and developed with imaging plates (BAS-IP MS 2340, Fujifilm, Japan). **E, F** To discriminate between MEK1 and MEK2, the unique peptides for each protein found during mass spectrometry were analyzed. There was a higher coverage (**E**) and intensity (**F**) for MEK1 compared to MEK2. **G** Predominant binding of MEK1 and not MEK2 shown by mass spectrometry analysis of peptide amounts and peptide coverage was confirmed with IPs enriching MACC1 from SW480/MACC1, SW620, and HEK293T/MACC1 cells. MEK1 and MEK2 were detected during immunoblotting with monoclonal antibodies specific for one isoform only from the same IPs.

Next, we aimed to integrate MACC1 in MAPK signaling. We determined the MACC1 interactome and analyzed it specifically for interacting protein kinases. First we characterized the specificity of the antibodies intended for IP by MS. Both antibodies were pulling-down MACC1 confirmed by shot-gun MS. MACC1 specificity of the interactome was demonstrated by identifying MACC1-specific peptides in the IP experiment (Fig. 2A).

We performed independent MACC1 IPs from SW620 cells with high endogenous MACC1 and subjected the eluates to quantitative proteomic analysis (Fig. 2B). Twenty-two out of 1203 interacting proteins were kinases (Fig. 2B), with 21 belonging to the serine/threonine kinase family [34]. The kinase with highest affinity was MEK1 (MAP2K1; dual specificity MAP kinase kinase 1), member of the very small family of dual specificity protein kinases phosphorylating substrates at tyrosine and subsequently at threonine residues [34]. With MEK1 being the strongest interactor, we confirmed the MEK1–MACC1 interaction by IP in SW620 cells already used for previous MS, and in SW480/MACC1-wt cells (Fig. 2C).

We applied recombinant constitutively active MEK1 (S218D, S222D) to MACC1-GFP isolated from HEK293T cells using in vitro γP32 ATP kinase assay. MEK1 (S218D, S222D) rapidly phosphorylated MACC1-GFP leading to a detectable signal after 15 min, with a maximum phosphorylation after 45 min. This transfer of radioactive phosphate was strongly reduced by excess of cold ATP, showing the specificity of the phosphorylation assay. Importantly, this reaction was inhibited by treatment with the MEK1 inhibitor AZD6244 (Fig. 2D).

MEK1 was found with higher coverage compared to MEK2 (seven vs. four unique peptides, 112/393 (28.5%) vs. 54/400 (13.5%) amino acid residues, respectively) and fourfold higher intensity in SW480/MACC1 and SW620 cells (Fig. 2E, F). To validate the interaction of primarily MEK1 and not MEK2, we applied SW480, SW620, and HEK293T cells with high MACC1 expression in IP experiments (Fig. 2G). Using the very same IPs, we found only very low levels of MEK2 interacting with MACC1 compared to MEK1, although both kinases are readily expressed in these cells.

Taken together, we demonstrated by independent approaches that MACC1 is a new target for MEK1. MEK1 interacts with MACC1 that becomes subsequently phosphorylated.

### MACC1 and MEK1 expression in human CRC specimens

**Fig. 3:**
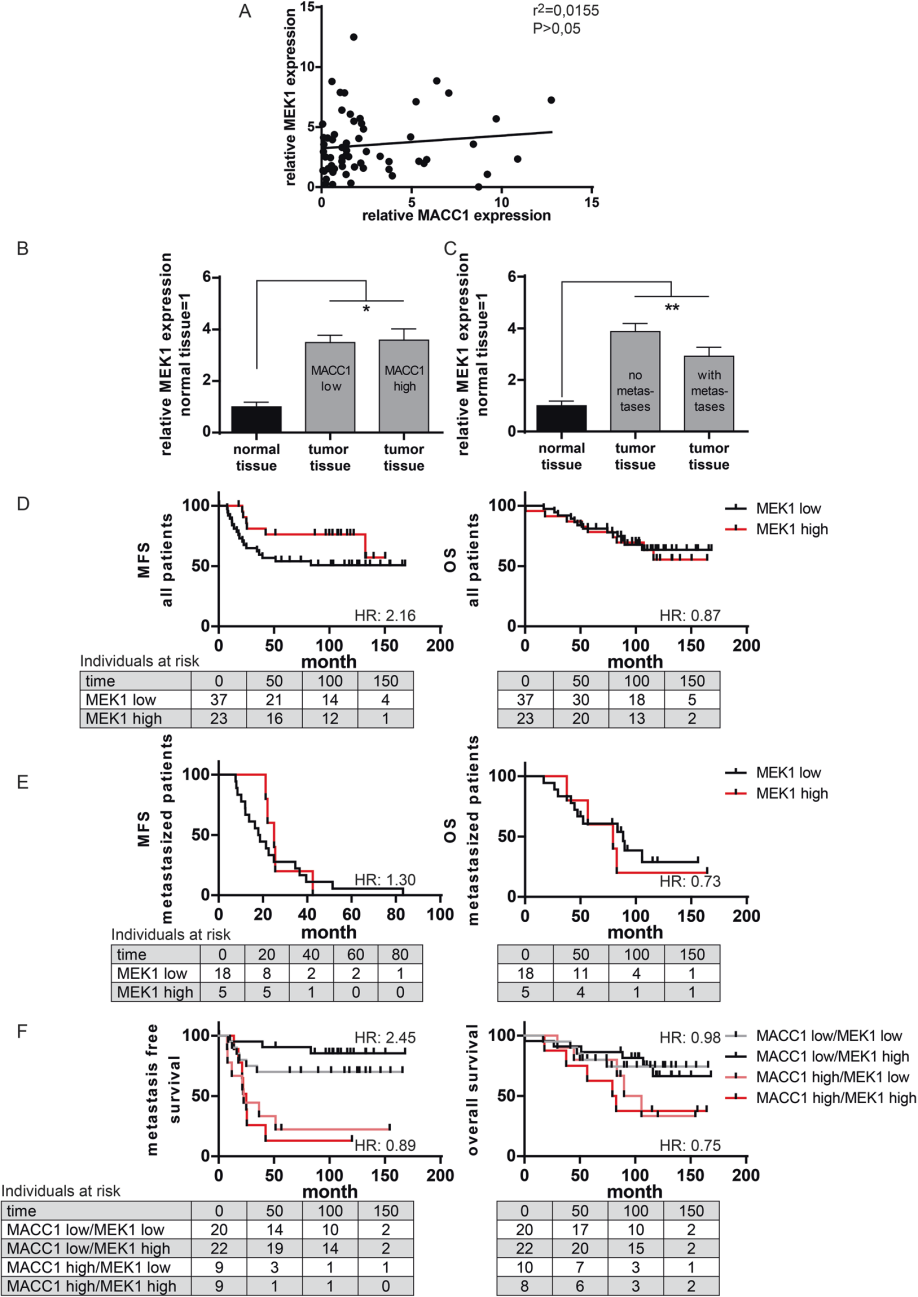
MEK1 mRNA expression level in clinical tumor samples is not associated with survival. A cohort of 60 stage I–III colon cancer patients was analyzed. The patients did not receive prior treatment and were not distantly metastasized at time of diagnosis. **A** Analysis revealed no correlation of MEK1 and MACC1 mRNA expression in the complete cohort of 60 patients (stage I–III). **B** Analysis of all 60 patients grouped for low or high MACC1 mRNA expression, showed no difference in the expression of MEK1 mRNA in the tumor tissue. In contrast, there is a statistically significant increase of MEK1 mRNA expression compared to normal mucosa. **C** Analysis of all 60 patients grouped for metastasis formation, determined no difference in MEK1 mRNA expression between these two groups. However, both patient cohorts show an elevated MEK1 mRNA expression compared to normal mucosa. **D–F** Survival analysis of colon cancer patients. Analysis of metastasis-free or overall survival in all (*N* = 60 patients) **(D)** or the later, metachronously metastasizing patients (*N* = 23 out of 60 patients) **(E)** revealed no association with MEK1 mRNA expression level. Subdivision of all patients (*N* = 60) in MACC1 mRNA low and high groups, does not improve prognosis for metastasis-free and overall survival by inclusion of MEK1 mRNA expression **(F)**. The curves are not statistically significant different from each other. Below each survival curve the individuals at risk are indicated.

Finally, to assess if MEK1 is available in tumor tissue, we used the cohort of CRC patients from the initial discovery of MACC1 [4] for MEK1 expression analysis as well as samples from normal mucosa. MEK1 is more than three times higher expressed in tumor tissue compared to normal colon tissue. However, there is no correlation of MACC1 and MEK1 expression in the cancer tissue of these patients (Fig. 3A). In contrast to MACC1 expression, MEK1 expression is not prognostic for occurrence of later metastasis, and is independent of tumor stage (Fig. 3B, C). Using a ROC-based cutoff for MEK1 low or high expression, there is no change in metastasis-free or overall survival of patients with high MEK1 expression compared to low MEK1 expressers (Fig. 3D). To analyze the impact of MEK1 gene expression specifically on survival time of the later metastasizing patients, we assessed the metachronously metastasizing patients only, excluding the not metastasizing patients. Again there is no difference in survival in dependency of MEK1 expression level (Fig. 3E). In addition, combining MEK1 expression with MACC1 expression in the survival analysis is not able to improve prognosis on MACC1-dependent metastasis-free or overall survival (Fig. 3F).

Thus, MEK1 is readily available in patient samples driving proliferation of tumor tissue, but expression level of the kinase MEK1 is not as important as that of its substrate MACC1 to force cancer cells to metastatic behavior. However, although MEK1 levels are not of prognostic relevance for CRC patients, its expression was found indispensable for MACC1-induced metastasis.

### The MACC1 interactome shows enrichment for MAP kinase signaling components

**Fig. 4:**
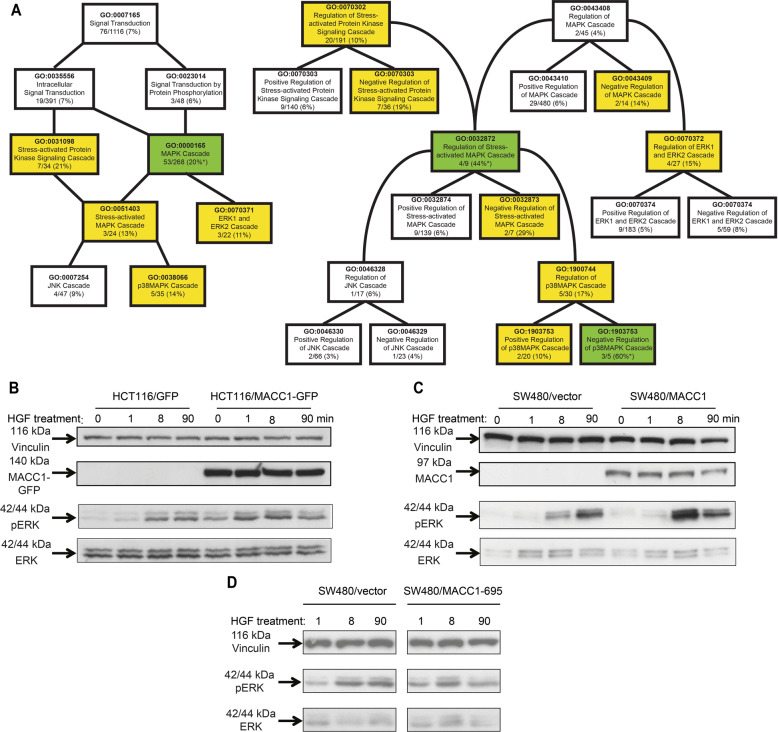
The MACC1 interactome is enriched for MAP kinase signaling proteins leading to increased signaling after HGF stimulation. **A** MACC1 interacts with proteins connected to MAPK signaling pathway cascades and their regulating complexes. The relations of individual GO terms focused on signal transduction and protein kinase cascades (left panel) and their corresponding regulation (right panel) are represented in black lines. The percentage of elements of each GO term that are interacting with MACC1 is indicated in %. A value >10% is highlighted in yellow. When both applies, a value is >10% and significantly different, the values are highlighted in green. **B, C** HGF induces ERK phosphorylation earlier and stronger when MACC1 is overexpressed. HCT116 **(B)** and SW480 **(C)** stably expressing the empty vector or MACC1 were treated with 20 U HGF. Similarly, SW480 stably transfected with MACC1-695 were treated with HGF but failed to increase pERK compared to the vector control (**D**). Cells were harvested before (0 min) and after 1, 8, and 90 min of HGF treatment. Lysis was performed with RIPA buffer supplemented with protease and phosphatase inhibitors. Equal amounts were separated with polyacrylamide gels and vinculin (loading control), ERK, pERK, and MACC1 (SW480) or MACC1-GFP (HCT116) were detected. ERK phosphorylation is induced in MACC1 overexpressing HCT116 and SW480 cells upon treatment with HGF more than threefold (SW480) and twofold (HCT116) within the first 8 min of HGF treatment. This induction is not evident if MACC1-695 is overexpressed in SW480.

We analyzed the MACC1 interactome to identify protein interactions critical for MAPK signaling and potentially leading to functional modulations of MACC1, which might be essential for the metastasis-inducing role of the protein. We analyzed the 1203 potential interactors of MACC1 for enrichment of GO terms linked to cellular response to extrinsic factors and associated signal transduction pathways. We found enrichment of MACC1 interactors associated with the MAP kinase cascade in general (52/266, *p* < 0.05), as well as in GO terms differentiating the MAP kinase pathways (Fig. 4A).

We tested for an increased MAP kinase pathway activity by assessing ERK phosphorylation as final effector of activated MAP kinase signaling. After stimulation of MACC1 overexpressing cells with HGF, ERK activation was increased compared to cells without ectopic MACC1 overexpression (Fig. 4B, C). This induction is not evident if MACC1-695 is overexpressed in SW480 (Fig. 4D).

In summary, MACC1 is part of MAP kinase signaling pathway leading to increased and accelerated ERK activation after HGF stimulation.

### Identification of MACC1 tyrosine phosphorylation sites

**Fig. 5:**
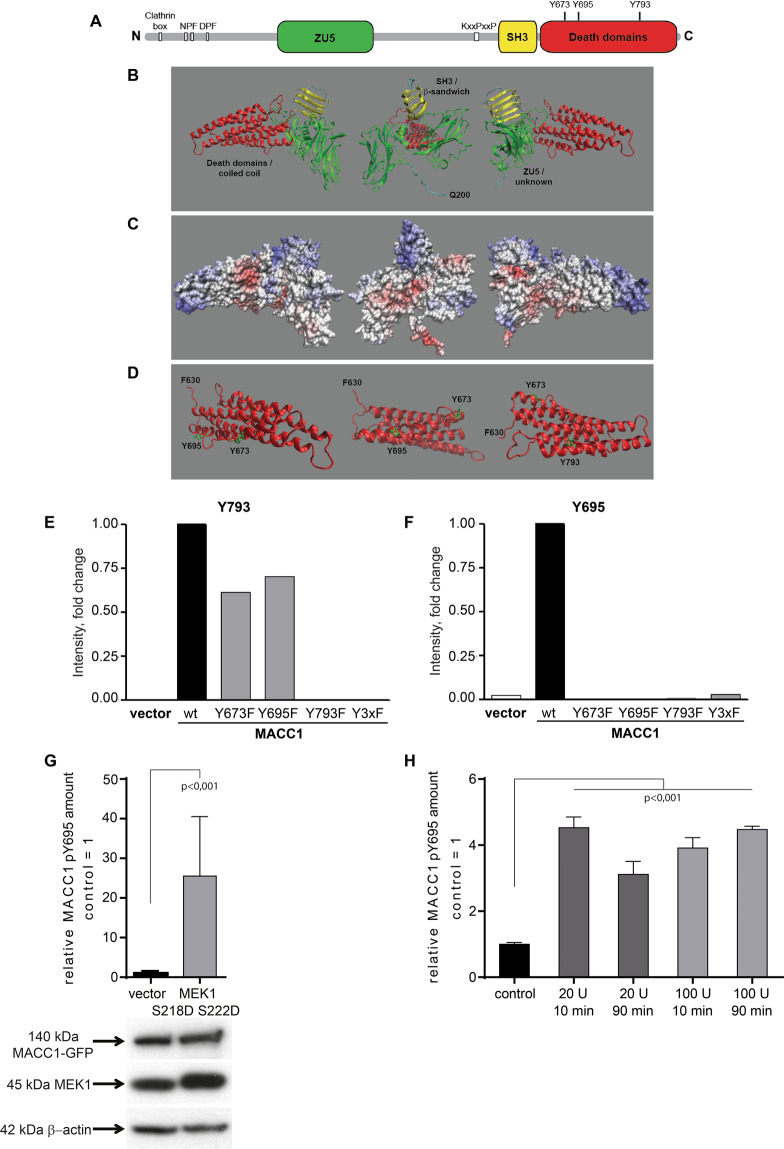
Predicted domain architecture, folding and tyrosine phosphorylation sites of MACC1. **A** Overview of structural domains (colored boxes) and interaction motifs (white boxes) predicted by sequence alignment. ZU5 (green), SH3 (yellow), variant Src homology 3 domain; DD (red), death domain; NPF, Epsin homology 15 interacting motif; DPF, adaptor protein 2α interacting motif; KxxPxxP, proline-rich motif, class I SH3 interaction motif. **B, C** Predicted folding of MACC1, according to sequence alignment, comparison with known structures and modeling of highly similar domains. The N-terminus (aa 1–199) of MACC1 was predicted to be highly disordered and was omitted in the representation. Backbone view **(B)** and space filling representation with electrostatic charge distribution **(C)** are displayed from three different angles. Backbone representation **(B)**: ß-sandwich structure overlapping with the predicted SH3 domain (yellow); 2 adjacent similar folds overlapping the predicted ZU5 domain (green); coiled coil structure overlapping with both predicted death domains (red). Space filling representation **(C)**: negatively charged/acidic areas (red); positively charged/basic patches (blue); neutral/hydrophobic areas (white). **D** Position of three tyrosine residues (Y673, Y695, Y793) within the coiled coil structure, which are predicted sites for tyrosine phosphorylation. All three tyrosine residues are predicted to face the solvent and are accessible for modifying enzymes. Backbone representation of the coiled coil structure (red); tyrosine residues in licorice representation (green). **E, F** Quantification of the MACC1 tyrosine phosphorylation pattern was performed by SRM-based MS. Phosphorylation of tyrosine residue Y793 **(E)** and Y695 **(F)** was determined in SW480/vector, SW480/MACC1-wt, SW480/Y673AF, SW480/Y693F, SW480/Y793F, and SW480/Y3xF cells. SDM of the sites Y793 and Y695 of MACC1 restricted phosphorylation. Phosphorylation of position Y793 was independent of phosphorylation at Y673 and Y695, whereas phosphorylation of Y695 was dependent on phosphorylation at positions Y673 and Y793 demonstrating a hierarchy of phosphorylation events. **G** Ectopic overexpression of constitutively active MEK1 increases phosphorylation of MACC1 (peptide VSpYVIK) in HEK293T cells. MS was performed three independent times, with three technical replicates in each experiment. Given are the relative amounts of MACC1 peptide VSpYVIK compared to control cells, expressed as mean with SD. Western Blot analyses confirm MEK1 S218D S222D overexpression. **H** To analyze the impact of HGF treatment on phosphorylation of endogenous MACC1, SW620 cells with endogenous MEK1 expression and high endogenous MACC1 level were treated with HGF. Cells were serum starved for 4 h and subjected to 20 or 100 U of HGF. After indicated incubation times cells were harvested and lysed in RIPA buffer supplemented with phosphatase and protease inhibitors. MACC1 was isolated using IP. Amounts of phosphorylated MACC1 (peptide VSpYVIK) were quantified with SRM-MS. All treatments led to significantly increases in pY MACC1. Values are shown relative to control cells without HGF treatment with SD. Significance was analyzed using ANOVA with the Dunnett’s multiple comparison test.

Tyrosine phosphorylation is of crucial importance in cellular signaling processes. Since MEK1 is the only kinase of the MACC1 kinome, which is able to phosphorylate tyrosine residues, we searched for potential MACC1 tyrosine phosphorylation sites using PROSCAN. We identified Y673, KVLADVLG**Y** (amino acids (aa) 665–673); Y695, KESEKVS**Y** (aa 688–695), Y793, KPAYDFL**Y** (aa 786–793) (Prosite access: PS0007, Prosite documentation access: PDOC0007, [RK]-x(2,3)-[DE]-x(2,3)-Y; Randomized probability: min = 4.074e − 04 max = 4.083e − 04; Fig. 5A and Table S1).

We visualized these tyrosine phosphorylation sites in a predicted MACC1 3D structure, generated by the protein structure prediction service PHYRE2 [35]. In silico predictions, based on the MACC1 protein sequence, show motifs for various interaction partners and sites of post-translational modifications, predestining it as a signal transducer [16, 24, 36]. The N-terminal part of MACC1 contains linear motifs and domains for interaction with different proteins. More C-terminal, a proline-rich motif for Src homology 3 (SH3) domain interaction is followed by an SH3 domain, and adjacent death domains (Fig. 5A–D).

Modeling the MACC1 3D structure based on sequence alignments resulted in a compact protein structure with globular domains. Due to the intrinsic disorder profile, the N-terminal sequence (aa 1–199) of MACC1 and a more central sequence (aa 518–556) were not modeled and are missing in the visualized 3D structure. The predicted ZU5 domain is similar to Ankyrin. The variant SH3 domain of MACC1 is predicted as a compact fold of parallel β-strands. Two predicted, partially overlapping death domains are suggested as one large coiled coil domain (Fig. 5B). Surface charge distribution was calculated according to the predicted localization of charged residues and was mapped onto the protein surface (Fig. 5C). Tyrosine residues Y673, Y695, and Y793, which have a high probability to be phosphorylated, are located within the predicted death domains. Importantly, they face the surface of the coiled coil structure, rendering them accessible to tyrosine kinases (Fig. 5D). Animations of the predicted protein structure of MACC1 and its C-terminal death domains with highlighted tyrosine residues can be found in Movies S1 and S2, respectively.

To isolate the impact of each predicted tyrosine phosphorylation site, we performed site-directed mutagenesis (SDM) substituting tyrosine by phenylalanine (Fig. S2A and Table S2), generating the MACC1 mutants Y673F, Y695F, Y793F, and a triple mutant harboring all three mutations, Y3xF. By this, the calculated local electrostatic surface charge distributions were not changed (Fig. S2B). SDM was confirmed by sequencing (Fig. S2C). SW480 cells were transfected for stable overexpression of MACC1 pY mutants, generating SW480/MACC1-Y673F, SW480/MACC1-Y695F, SW480/MACC1-Y793F, and SW480/MACC1-Y3xF for functional experiments, (additionally to SW480/vector and SW480/MACC1-wt) (Fig. S2D).

### MACC1 tyrosine phosphorylation is hierarchical and inducible by HGF

To prove the phosphorylation of MACC1 at the predicted tyrosine residues and their disappearance following SDM, targeted proteomics was used. Characteristic fragment ions of pY-containing peptides were followed by SRM-MS. We found elevated Y793 phosphorylation levels in SW480/MACC1-wt, SW480/MACC1-Y673F, and SW480/MACC1-Y695F cells (Fig. 5E). We observed no Y793 phosphorylation in SW480/MACC1-Y793F and SW480/MACC1-Y3xF cells. This indicates the SDM-caused restriction of the Y793 phosphorylation. The phosphorylation of Y793 is independent of the phosphorylation of Y673 and Y695. Y793 is still phosphorylated when Y673 or Y695 is missing.

Phosphorylation of Y695, found in SW480/MACC1-wt cells (Fig. 5F), was absent in SW480/MACC1-Y695F cells, verifying that SDM of tyrosine Y695 to phenylalanine abolished tyrosine phosphorylation at this particular site. Surprisingly, Y695 phosphorylation was lost in all pY mutants. This finding indicates, in addition, the dependence of Y695 phosphorylation on the phosphorylation of both, Y673 and Y793. This clearly points to a hierarchy of MACC1 tyrosine phosphorylation with initial modifications of Y673 and Y793 as prerequisites for the phosphorylation of Y695. The peptide harboring Y673 was ionizing poorly and was therefore not detected in the analysis of total cell extracts.

To validate MEK1 as the kinase phosphorylating MACC1, we overexpressed MACC1-GFP and constitutively active MEK1 (S218D, S222D) in HEK293T cells. SRM-MS of pY MACC1 confirmed that MEK1 overexpression leads to higher MACC1 phosphorylation levels. MACC1 pY-position 695 gets 25-fold more phosphorylated (*p* < 0.001) compared to control cells without MEK1 overexpression (Fig. 5G). Reciprocally, after stable downregulation of MEK1 by shRNA in MACC1-GFP overexpressing HEK293T, RKO and SW480 cells, MACC1 phosphorylation at pY-site 695 was reduced compared to control cells (Fig. S3A–C).

In a physiologic situation, MEK1 needs to be activated by growth factors like HGF. HGF (scatter factor) is the basis for cell dissemination phenotype in SW480/MACC1 cells [4]. To use a cellular system without ectopic overexpression, we treated endogenously high MACC1-expressing SW620 cells with different amounts of HGF for 10 and 90 min. HGF induced MACC1 phosphorylation at position Y695 at both concentrations and time points (Fig. 5H).

In conclusion, phosphorylation of these MACC1 tyrosine residues occurs at predicted sites, and in a hierarchical manner.

### MACC1 tyrosine phosphorylation is critical for target gene MET expression, motility, and proliferation of CRC cells

**Fig. 6:**
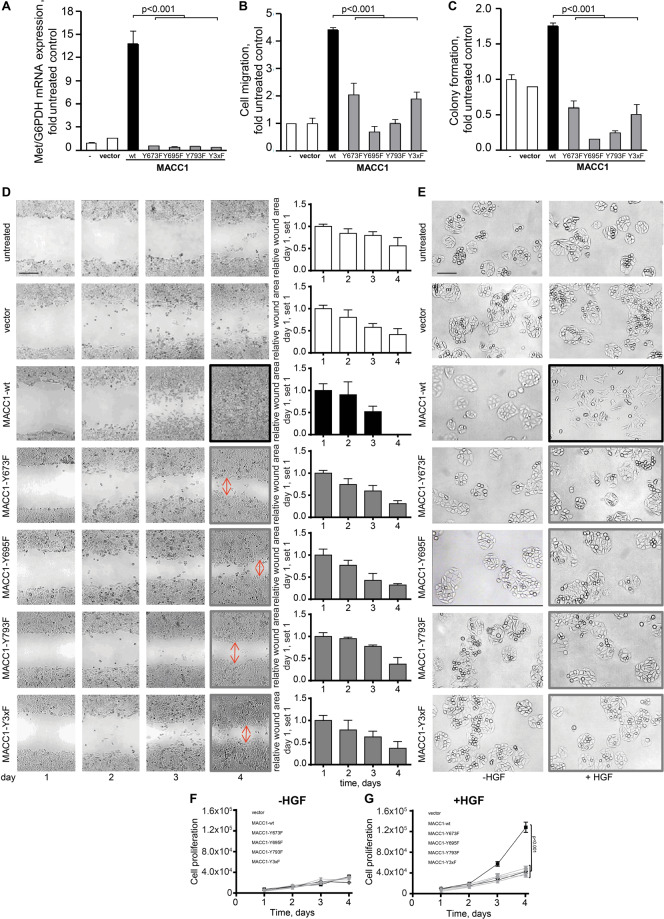
Mutation of MACC1 tyrosine phosphorylation sites restricts MET expression and MACC1-induced in vitro cell motility and proliferation. **A–C** SDM of the tyrosine phosphorylation sites Y673, Y695, and Y793 restricts MACC1-wt-induced expression of its transcriptional target gene MET **(A)**. SDM of the tyrosine phosphorylation sites Y673, Y695, and Y793 restricts MACC1-wt-induced cell migration **(B)** and MACC1-wt-induced colony formation **(C)**. For all comparisons: SW480/MACC1-wt vs. each of the MACC1 pY mutants: all *p* < 0.001. Data are presented as mean with SD. **D** SDM of the tyrosine phosphorylation sites Y673, Y695, and Y793 restricts MACC1-wt-induced wound healing. Complete wound closure was documented at day 4 for SW480/MACC1-wt cells, whereas clearly visible wounds were seen in all MACC1-pY mutants cell monolayers. Quantification of wound area over time was done compared to day 1. Scale bar, 200 µm. Data are presented as mean with SD. **E** SDM of the tyrosine phosphorylation sites Y673, Y695, and Y793 restricts MACC1-wt-induced cell scattering. HGF-induction of cell scattering in SW480/MACC1-wt cells was not observed in the MACC1-pY mutants. Scale bar, 50 µm. **F, G** SDM of the tyrosine phosphorylation sites Y673, Y695, and Y793 restricts MACC1-wt-induced HGF-mediated cell proliferation. For all comparisons: SW480/MACC1-wt vs. each of the MACC1 pY mutants: all *p* < 0.001. Data are presented as mean with SD.

We analyzed the functional impact of the MACC1 tyrosine mutations on expression of the receptor tyrosine kinase MET, one of its key transcriptional target genes [4, 37]. MET expression was lower in all MACC1 pY mutants (vs. SW480/MACC1-wt; all *p* < 0.001) resembling SW480 and SW480/vector levels (Fig. 6A). This is strongly indicating the functional importance of MACC1 tyrosine phosphorylation.

Next, we addressed the pY-dependent MACC1 motility- and proliferation-inducing abilities. All pY mutants reduced the number of migrated cells (vs. SW480/MACC1-wt; all *p* < 0.001; Fig. 6B), and of colonies (vs. SW480/MACC1-wt; all *p* < 0.001; Fig. 6C). Complete wound healing was observed at day 4 for SW480/MACC1-wt cells, but was delayed in all pY mutants, SW480 and SW480/vector cells (Fig. 6D). HGF-induced cell dissemination (scattering) was seen in SW480/MACC1-wt cells, but neither in pY mutants nor in SW480 and SW480/vector controls (Fig. 6E). HGF-induced proliferation was exclusively observed in SW480/MACC1-wt cells, vs. pY mutants, SW480/vector and all untreated cells (all *p* < 0.001; Fig. 6F, G).

Taken together, tyrosine phosphorylation of MACC1 is essential for MACC1-induced cell motility and proliferation. Mutating the MACC1 tyrosine phosphorylation sites, results in loss of these MACC1-induced phenotypes.

### MACC1 tyrosine phosphorylation is essential for MACC1-induced CRC metastasis in mice

**Fig. 7:**
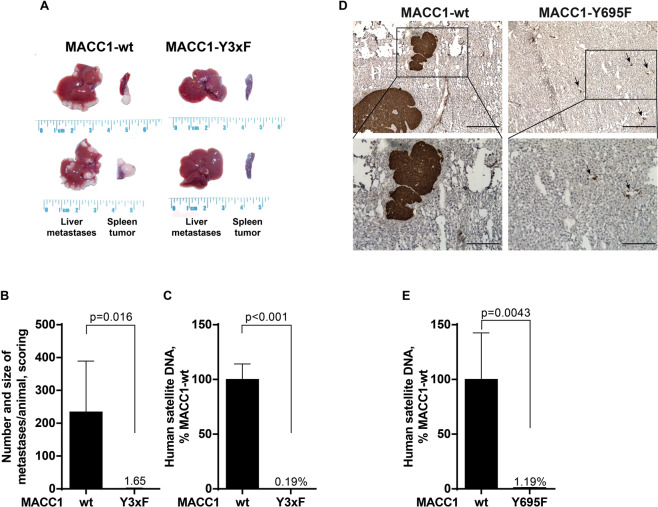
Mutation of MACC1 tyrosine phosphorylation sites restricts MACC1-induced metastasis in mice. SW480/MACC1-wt, SW480/MACC1-Y3xF, or SW480/MACC1-Y695F cells were injected into the spleen of mice. At the experimental endpoint, anesthetized mice were sacrificed and organs (spleen, site of transplantation; liver, site of metastasis) were removed. **A** Transplantation of SW480/MACC1-wt cells induced tumor growth in the spleens and liver metastasis in vivo. SDM of the tyrosine phosphorylation sites Y673, Y695, and Y793 restricts MACC1-induced tumor growth in the spleens and liver metastasis in the SW480/MACC1-Y3xF-transplanted animals. Isolated organs are shown for two representative animals per group with *n* = 16 animals per group. **B** Metastasis formation in the livers was quantified by scoring, considering number and size of the metastases. Mice transplanted with SW480/MACC1-Y3xF cells developed significantly less and smaller metastases compared to SW480/MACC1-wt mice (*p* = 0.016). **C** Metastasis formation in the livers was quantified by human satellite qPCR. Mice transplanted with SW480/MACC1-Y3xF cells showed significantly less human DNA in the liver tissue (*p* < 0.001) compared to SW480/MACC1-wt mice. **D** Metastasis formation in the liver of animals (*N* = 10) xenotransplanted with cells overexpressing Y695F-mutated MACC1 was visualized with staining of human CK19. Transplantation of SW480/MACC1-wt cells results in larger metastases compared to mice transplanted with SW480/MACC1-Y695F cells. Scale bars: 400 µm (upper panel, ×10 magnification) or 200 µm (lower panel, ×20 magnification). **E** Metastasis formation in the livers was quantified by human satellite qPCR. Mice transplanted with SW480/MACC1-Y695F cells showed significantly less human DNA in the liver tissue (*p* = 0.0043) compared to SW480/MACC1-wt mice. Data are presented as mean with SEM. Significance was calculated using the Mann–Whitney *t*-test.

We addressed the impact of the pY sites for MACC1-induced metastasis in NOD-SCID mice, intrasplenically injected with SW480/MACC1-wt or SW480/MACC1-Y3xF cells (Experimental Pharmacology & Oncology GmbH, Berlin). Tumor growth and liver metastases were induced in SW480/MACC1-wt-transplanted mice, confirming our previous findings [4, 6]. Remarkably, the MACC1-induced metastasis was reduced in MACC1-Y3xF-transplanted mice (Fig. 7A).

MACC1-Y3xF mice developed much less and much smaller liver metastases (vs. MACC1-wt mice; *p* = 0.016, Fig. 7B). In livers from MACC1-Y3xF mice, the amount of human cells quantified by human satellite DNA [38] was reduced to 0.19% (vs. MACC1-wt mice; set 100%; *p* < 0.001; Fig. 7C) again indicating the restriction of liver metastasis by Y3xF-mutant MACC1.

We confirmed the importance of the single phosphorylation site Y695 by transplanting MACC1-Y695F overexpressing SW480 cells into mice. Of note, metastasis formation was also strongly reduced compared to SW480-MACC-wt overexpressing cells, even if only one specific amino acid residue was changed (Fig. 7D, E).

Thus, MACC1 tyrosine phosphorylation sites Y673, Y695, and Y793 are indispensable for MACC1-induced in vivo metastasis. Mutating the MACC1 tyrosine phosphorylation sites, results in diminished tumor growth and metastasis in mice.

## Discussion

Here we report for the first time the ability of clinically relevant MEK1 inhibitors to restrict MACC1-induced cell migration, tumor growth, and most importantly, metastasis in mice. MEK1 together with further MAP kinase signaling components are enriched in the MACC1 interactome. The dual specificity protein kinase MEK1 is the only kinase within the MACC1 kinome capable of tyrosine phosphorylation, defining MACC1 for the first time as novel substrate for MEK1 tyrosine phosphorylation. Tyrosine phosphorylation of MACC1 led to accelerated and increased activity of this pathway after HGF stimulation. We identified three important pY positions by in silico methods. Using SRM-MS, we showed the interdependence of the pY sites pointing to a hierarchical order of their phosphorylation. Stimulated or constitutively activated MEK1 led to increased levels of phosphorylated MACC1. Lacking these pY sites restricted MACC1-induced motility and proliferation in cell culture and metastasis in mice demonstrating their functional impact.

Taken together, MEK1-mediated MACC1 tyrosine phosphorylation is essential for MACC1 function regarding to tumor growth and metastasis, and is druggable by MEK1 inhibitors to restrict tumor growth and metastasis.

Using shot-gun proteomics, we identified the MACC1 interactome comprising 1203 potential protein partners. Among 22 kinases, only MEK1 phosphorylates tyrosine residues. MEK1, belonging to the small group of dual specificity kinases catalyzing tyrosine and threonine phosphorylation, is a decisive MAPK pathway component, activated by upstream regulators (ligand/cell surface receptors, Ras, Raf). MEK1 mediates signals to ERK via phosphorylation of the T-E-Y motif [39]. Activated ERK in turn exerts numerous molecular functions in both, the cytoplasm and the nucleus [40]. For binding of MEK1 to other proteins, the proline-rich sequences of MEK1 are required, which are able to bind SH3 domains [41, 42]. Both, a proline-rich sequence and an SH3 domain, are present in MACC1, hypothesizing a MEK1–MACC1-interaction mechanism [43].

The MAP-kinase kinase MEK1 can phosphorylate threonine and tyrosine residues [44]. It is paradigmed, that ERK is the only physiological substrate for MEK1 [45]. This view was challenged recently by Tang et al. [46]. They report on HSF1 as new target for MEK1. We demonstrate the metastasis inducer MACC1 as another novel target for MEK1, thereby providing a new link from accelerated MAP kinase signaling to metastasis. This broadens the view, that signaling through MEK is not linear leading to ERK activation, but branches at this point toward different effector molecules. This opens the possibility, that different signals are processed via MEK to regulate the respective outcome, like proteomic stability [46], motility [47, 48], or survival [49–51]. MACC1 facilitates this process by accelerating and increasing ERK activation after ligand binding. Here we have shown in vitro and in vivo, that MEK1 activity is necessary to activate MACC1 via phosphorylation. This happens in a hierarchical fashion through phosphorylation first at tyrosine 673 and 793 followed by tyrosine at position 695. This in turn leads to MET expression and sustained signaling through at least ERK that results ultimately in metastasis formation. However, although our study focuses on MEK1, this does not exclude other kinases involved in the regulation of MACC1 activity.

In our initial publication discovering the gene MACC1 we considered pathways MACC1 might be involved in using inhibitors for MAPK and PI3K signaling [4]. Scattering, exclusively observed in MACC1-high expressing cells, was completely restricted by employing two MEK1 inhibitors, but not by PI3K inhibitors. This was indicating that the MAPK pathway might be crucial for MACC1 action. Now we have identified this key molecule, MEK1, as MACC1 binding partner bringing both molecules into functional context.

The MAP kinase pathway is commonly activated in numerous cancers. It is therefore an attractive therapeutic target [52]. In our CRC patient, cohort MEK1 is expressed at elevated levels in all tissue samples compared to normal tissue controls. MEK1 expression is not able to improve prognosis for MACC1-dependent metastasis, metastasis-free, or overall survival. If MACC1 is expressed by the tumor cells, we found ERK activation increased and prolonged after stimulation. The strength and duration of the ERK signal are important to determine cell fate and cellular responses including proliferation, survival, or motility [45, 53, 54]. In this context, MEK1 phosphorylation of MACC1 is important to trigger its activation. To force cancer cells to metastatic behavior, MACC1 and MEK1 have to be co-expressed with the level of MACC1 as decisive inducer of metastasis.

Since the first description of small molecule inhibitors of MEK several compounds was developed for this purpose including the clinically used drugs AZD6244 (ARRY142886, selumetinib) and GSK1120212 (JTP74057, trametinib) [55, 56]. Both inhibit metastasis in mouse xenografts. Phase II trials evaluating AZD6244 as monotherapy or in combinations were conducted in patients with different cancer types (www.clinicaltrials.gov). GSK1120212 is the first MEK inhibitor licensed for advanced melanoma [57, 58]. It is used in phase II and III trials for patients with different cancer types (www.clinicaltrials.gov). Thereby, progression-free survival was prolonged, biomarkers for higher response rates were identified, or treatment regimens combined with chemotherapeutic drugs or further small molecule inhibitors were found to be more efficient [59, 60]. Since tyrosine phosphorylation is indispensable for MACC1-induced motility, proliferation, and metastasis in mice, and MEK1 is the only kinase of the MACC1 interactome able to phosphorylate tyrosine, we evaluated clinically applied MEK1 inhibitors. Remarkably, tumor growth and metastasis in mice exclusively attributed to ectopic MACC1 overexpression were restricted by AZD6244 and GSK1120212. AZD6244 also inhibited growth of xenografted tumors from endogenously high MACC1-expressing SW620 and HT29 cells [33, 61], but not in animals xenografted with endogenously low MACC1-expressing HCT15 or SW480 CRC cells [62]. GSK1120212 showed antitumor effects in highly MACC1-expressing HT29-xenografted animals [63]. Further MEK inhibitors are in preclinical and clinical development [64, 65]. Thus, we hypothesize MACC1 as new promising predictor for MEK1 inhibitor response.

MACC1-induced migration in vitro was lowered by all MEK1 inhibitors used. During clinical application of MEK1 inhibitors like selumetinib and trametinib, tumor cells may develop resistance toward these drugs. Therefore, this group of drugs is likely combined with other molecules [66–69]. Bernards et al. have shown a possible feedback loop via MAP3K1 or MAP2K4 leading to JNK/Jun pathway activation and cellular survival [70]. They expect a synergistic activity of (not available yet) MAP3K1 or MAP2K4 inhibitors and MEK1 inhibitors. Since MACC1 expression leading to cell survival and metastasis is based at least in part on c-Jun activity, application of a c-Jun inhibiting statins (e.g., lovastatin) might fill this gap. Cell motility should be synergistically reduced by combining a MEK1 inhibitor (e.g., AZD6244) and the MACC1 gene expression targeting lovastatin [27]. The usefulness of this combination was recently predicted through analysis of the NCI-60 cell line panel and in publically available databases [71].

Following our initial discovery of the novel gene MACC1 [4], transcriptional and post-transcriptional MACC1 regulations were reported [24–27, 72]. However, the importance of MACC1 post-translational modifications like tyrosine phosphorylation and the underlying signaling network are not yet investigated. Here we focused on predicted pY positions Y673, Y695, and Y793. We used SRM-MS to quantify tyrosine phosphorylation [73]. While Y793 phosphorylation is independent of Y673 and Y695 phosphorylation, Y695 phosphorylation depends on both Y673 and Y793 sites, suggesting a critical sequence of MACC1 tyrosine phosphorylation. Sequential phosphorylation is known for key signal transduction molecules, e.g., c-myc, 4E-BP1, and β-catenin, initially phosphorylated by CK1 at S45, thereby priming phosphorylation by GSK3β at T41, S37, S33 [74].

We isolated the effect of each pY mutation in functional assays. MACC1-wt-induced MET expression, migration, colony formation and proliferation, wound closure, and scattering were restricted by each pY-mutated MACC1. Liver metastasis in mice, solely induced by ectopic MACC1-wt overexpression, was completely inhibited by the Y3xF mutant. Importance of wt-pY sites is underlined by our MACC1-SNP analysis of 154 human CRC, where none of the identified MACC1-SNPs interfered with the critical pY sites [75].

In summary, MACC1 tyrosine phosphorylation is decisive for switching from tumor growth to motility and metastasis. Targeting MACC1 tyrosine phosphorylation using MEK1 inhibitors opens new options to intervene in MACC1-induced cell proliferation, motility, and metastasis aiming at the ultimate goal of personalized tailored therapies for inhibition of cancer progression and metastasis, resulting in improved patient survival. Since MACC1 is confirmed as decisive driver for tumor growth and metastasis in a variety of solid cancers, the findings made here for CRC might be translated to further solid tumor types [17–20, 25]. The usefulness of MACC1 as therapeutic target toward MEK1 inhibitor treatment requires confirmation in clinical trials.

## Materials and methods

### Drugs and treatments

MEK1 inhibitors (UO126, PD98059: Cell Signaling Technology, Danvers, MA, USA; AZD6244 GSK1120212: Selleck Chemicals Munich, Germany) were solubilized in DMSO. For migration experiments, cells were treated with 20-µM UO126, 50-µM PD98059, 10-µM AZD6244, and 10-µM GSK1120212, respectively, for 24 h. DMSO-treated cells served as controls.

For in vivo application, 50-mg/kg AZD6244 was administered twice daily orally and 2-mg/kg GSK1120212 was given once daily orally (10% Kolliphor EL, Sigma Aldrich, Munich, Germany; 0.9% NaCl solution). Control mice were treated with the appropriate volume of 10% Kolliphor EL, 0.9% NaCl.

### Plasmids and site-directed mutagenesis

Point mutations to putative tyrosine (Y) phosphorylation sites Y673, Y695, and Y793 in MACC1 full-length cDNA cloned into pcDNA3.1D/V5-His-Topo (Thermo Fisher, Waltham, MA, USA) vector (pcDNA3.1D/MACC1) [4] were introduced by SDM (QuickChange Kit, Agilent, Santa Clara, CA, USA; primers: Tables S2 and S3, Biotez, Berlin, Germany). A triple pY-mutated construct was generated sequentially starting with Y673F as a template followed by introduction of Y695F and Y793F (Fig. S2A). Mutations were confirmed by sequencing (Fig. S2C).

### Cell lines and gene transfer

Human cell lines SW480, SW620, RKO, HCT116, and HEK293T (American Type Culture Collection, Manassas, VA, USA) were maintained at 37 °C in a humidified 5% carbon dioxide incubator in RPMI-1640 medium (SW480, HCT116) or DMEM (SW620, RKO, HEK293T) (Thermo Fisher) supplemented with 10% fetal bovine serum (FBS; Bio&Sell, Feucht, Germany). Cell lines were free of mycoplasma (MycoAlert Kit, Lonza, Basel, Switzerland). Authentication was performed by short tandem repeat genotyping at the DSMZ (German Collection of Microorganisms and Cell Cultures; Braunschweig, Germany).

Plasmids coding pY-mutated MACC1 were stably transfected (Lipofectin, Thermo Fisher) into SW480 cells and selected by G418 (Thermo Fisher). MACC1 expression was analyzed by quantitative real-time RT-PCR (qRT-PCR) and Western blotting. For each experiment, at least three independently transfected clones were analyzed; one representative clone thereof is shown.

For testing the ability of MEK1 inhibitors to restrict MACC1-induced metastasis in mice by bioluminescence, we employed SW480 cells stably transfected with pIRES/luc-MACC1 (pIRES: Takara, Mountain View, CA, USA).

MACC1-GFP, MEK1 shRNA, and constitutively active MEK1 were stably introduced using lentiviruses at a MOI of 5. MEK1 (S218D, S222D)-pcw107 was a gift from David Sabatini and Kris Wood (Addgene plasmid #64604, Cambridge, MA, USA), pcw107 was a gift from John Doench and David Sabatini (Addgene, plasmid #62511). MACC1-GFP containing plasmid was purchased from Origene (Rockville, MD, USA), the control plasmid was generated by in-frame removal of MACC1. MEK1 shRNA containing plasmids were obtained from BioCat (Heidelberg, Germany).

### In vitro migration assay

2.5 × 10^5^ cells in RPMI were seeded into 12-mm diameter-transwell upper chamber for 24 h (12-μm pore size, Merck, Darmstadt, Germany). Drug treatment was performed before seeding as indicated and continued in the upper and lower chamber. RPMI with 10% FBS was added to the bottom chamber. Migrated cells in the lower chamber were counted using Neubauer chambers (Roth, Karlsruhe, Germany). Cell migration assay was performed three times, each in duplicate counted multiple times.

### Restriction of MACC1-induced metastasis in mice

Experiments were performed in accordance with the United Kingdom Coordinated Committee on Cancer Research guidelines and approved by the responsible local authorities (State Office of Health and Social Affairs, Berlin, Germany). Sample size (animal number) was calculated to reach statistical significance if the following assumptions are met: *p* ≤ 0.05, power of 80%, effect size 0.7–0.9. Animals were randomly allocated to the treatment groups after cell inoculation. All tumor forming animals were included in the analysis. During the animal work, no blinding was performed.

For evaluating the impact of tyrosine phosphorylation for MACC1-induced metastasis in vivo, 6-week-old female NOD-SCID mice were randomly assigned to two groups and intresplenically transplanted with 3 × 106 SW480/MACC1-wt or SW480/MACC1-Y3xF cells (*n* = 16). Similarly, SW480/MACC1-Y695F cells (*n* = 10, vs. wt) were assessed. Animals were sacrificed by cervical dislocation at the experimental endpoint. Metastasis was evaluated by numbering and by scoring based on the calculation for each liver as the sum of the volumes of the individual metastases (length × width^2^ in mm^3^). To screen for human cells in mouse livers, organs were collected at the experimental endpoint.

For evaluating the impact of MEK1 inhibitors on MACC1-induced metastasis in vivo, 6-week-old female SCID beige mice (Charles River, Sulzfeld, Germany) were intrasplenically transplanted with 3 × 10^6^ SW480/luc-MACC1 cells and randomly assigned in three groups.

Control mice (*n* = 6 mice) were treated daily with 2-ml/kg solvent orally (10% Kolliphor EL, 0.9% NaCl). AZD6244 (50 mg/kg) was applied twice, GSK1120212 once daily p.o. Treatments started at the day of transplantation and were continued until the animals were sacrificed. After recovery, bioluminescence imaging (NightOwl LB 981 systems, Berthold Technologies, Bad Wildbad, Germany) was performed twice per week after anesthesia with isofluran (Abbott GmbH, Wiesbaden, Germany) using 150-mg/kg D-luciferin (Biosynth, Staad, Switzerland) in PBS. Animal experiments were terminated due to ethical reasons on day 39 by cervical dislocation in accordance with the local authorities. Tumor growth and metastasis were quantified with ImageJ 1.48k (NIH, Bethesda, MD). Livers were collected and snap frozen for molecular analysis.

Similarly, for the RKO-based xenograft mouse model SCID beige mice were used. 3 × 10^6^ genetically manipulated RKO cells were transplanted intrasplenically. Anaesthetized mice were sacrificed by cervical dislocation and livers were collected for molecular analysis.

### RNA and qRT-PCR, DNA and qPCR

RNA was isolated (TRIzol, Thermo Fisher). Two-step qRT-PCR was performed in duplicate [4]. Briefly, 50-ng total RNA was reversely transcribed (random hexamer primers, 10-mM MgCl_2_, 1 × PCR-buffer II, 250-µM pooled dNTPs, 1-U/µL RNAse inhibitor, 2.5-U/µL MuLV reverse transcriptase; Thermo Fisher) at 42 °C for 15 min, 95 °C for 5 min. Quantitative PCR was performed for 10 min at 95 °C and 45 cycles of 10 s at 95 °C, 30 s at 60 °C, 4 s at 72 °C (LightCycler system, Roche). Mean values in duplicate were calculated using standard curves and normalized to the housekeeping gene.

For detection of metastasized human cells in mouse livers, genomic DNA was isolated from mouse livers (DNA-RNA-Protein Extraction Kit, Roboklon, Berlin, Germany). Quantitative PCR was performed using 50-ng genomic DNA. Titration was performed with genomic DNA from spiked human/mouse cell populations. Primer sequences (BioTeZ and TIB MolBiol, Berlin, Germany) are summarized in Table S4.

### SRM-based quantification of the phosho-tyrosine containing peptides and shot-gun proteomics

For quantification of tyrosine phosphorylation by SRM-MS, immunoprecipitated protein samples were solubilized in Laemmli loading buffer and subjected to SDS-PAGE. The MACC1 band was processed using an automated HTS PAL system (CTC Analytics, Switzerland). Peptides were extracted, purified, and stored on reversed-phase (C_18_) StageTips [76]. Peptides were resuspended in (A) 0.1% formic acid/(B) 5% acetonitrile. Heavy-labeled (Lys 8) internal standard peptides (SpikeTides, JPT Inc., Berlin, Germany) were added at 25 fmol/µg of protein. Each injection comprised of 1 μg of total protein. Peptides were separated on a 20-cm reversed-phase column, 75-μm ID, (C_18_ Reprosil, 3 μm, Dr Maisch GmbH, Ammerbuch, Germany) using a gradient from 5 to 40% B in 90 min, and detected by a Q-Trap 4000 and 6500 (AB Sciex, Darmstadt, Germany).

SRM methods were established by determining relevant transitions for the heavy (Lys 8) analog. The interface heater temperature was 150 °C, curtain gas was 30 psi, and ion spray voltage 2400 V. Applied collision gas was set to “high.” Q1 and Q3 were operated at unit resolution. The declustering potential was 130 V. The entrance potential and collision exit potential were set as default. For the collision energy and time see Table S5. All measurements were performed in positive mode. SRM signals were quantified using the MultiQuant 3.0 software package (AB Sciex), the R-statistical software package and the ggplot2 module. For SRM transitions used for quantification of the phospho-tyrosine peptides see Table S5. Three technical replicates of two independent biological replicates were measured and analyzed.

For identification of the MACC1 interactome by MS (shot-gun proteomics), IP of SW620 cells with two polyclonal rabbit anti-human MACC1 antibodies (HPA020103, HPA020081, Sigma, St. Louis, USA, 2 µg) was performed four times independently.

Samples were eluted from the affinity beads using denaturing buffer (6-M urea, 2-M thiourea, 20-mM HEPES, pH 8.0, Sigma). Proteins were digested using endopeptidase LysC (Wako, Japan) and trypsin (Promega, Madison, WI, USA). Peptides were desalted using StageTips [76], resuspended in 3% trifluor acetic acid/5% acetonitrile buffer (Sigma, Merck) and separated on a reversed-phase column (20-cm length, 75-μm ID, 3-μm Reprosil-C18, Dr Maisch, gradient: 5–45% acetonitrile). Peptides were ionized on a Proxeon ion source and directly sprayed into the mass spectrometer (Q-Exactive, Thermo Fisher). The recorded spectra were analyzed using the MaxQuant software package (Version, 1.2.2.5) with fixed modifications set to carbamylation of cysteines and variable modifications set to phosphorylation of serine, threonine, and tyrosine, and methionine oxidation. The false-discovery rate was set to 1% on protein and peptide level. Statistical analysis of the data set was performed using the R-statistical software package.

### Protein extraction, Western blotting, and immunoprecipitation

Cells were lysed in RIPA buffer (50-mM Tris pH 7.5; 0.15-M NaCl; 1% NP40; 0.5% sodiumdeoxycholate) supplemented with complete protease inhibitor tablets (Roche). Proteins were separated using Bis-Tris gels and transferred to Hybond C Extra nitrocellulose membranes. Detection of V5-tagged MACC1 protein was performed with a direct HRP-labeled monoclonal mouse anti-human V5-specific antibody (Thermo Fisher, 46-0308), for MEK1 with a monoclonal mouse anti-human MEK1 antibody (Cell Signaling, and for β-tubulin with a monoclonal mouse anti-human β-tubulin antibody (BD, 556321). Protein bands were visualized with ECL reagent and CL-XPosure Films (Thermo Fisher Scientific).

To isolate GFP-tagged MACC1, the GFP Nanotrap kit (Chromotek, Planegg-Martinsried, Germany) after cell lysis with the supplied IP lysis buffer supplemented with cOmplete protease cocktail and phosSTOP phosphatase (Sigma Aldrich) inhibitors was used.

For the identification of the MACC1 interactome by MS, cleared lysates of 2 × 10^6^ SW620 cells in lysis buffer (20-mM Tris-HCl pH 7.5, 150-mM NaCl) were employed for IP. Two different anti-human MACC1 antibodies (HPA020103, HPA020081, Sigma Aldrich) were used for MACC1 pulldown in duplicate.

For detection of the physical binding of MACC1 and MEK1, cleared lysates of 5 × 10^6^ cells in 20-mM Tris-HCl pH 7.5, 150-mM NaCl, 0.1% NP40, 1-mM EDTA, 1% Triton-X-100 were used. Proteins were enriched using MACC1 antibody (HPA020103), β-tubulin antibody (BD Bioscience, San Jose, CA, USA), MEK1 antibody (antibodies H-8 and A-1, respectively, Santa Cruz), and Protein G Agarose beads (Life Technologies). After elution with 4 x SDS sample buffer (5 min, 95 °C), MEK1 and MEK2 were detected by Western blotting (antibodies H-8 and A-1, respectively, Santa Cruz).

ERK, pERK, and vinculin were detected with monoclonal antibodies (Cell Signaling 9102, 9101 and Sigma V9131, respectively).

### Kinase assay

Ten micrograms MACC1-GFP from Nanotrap IP were incubated with 500-ng recombinant MEK1 S218D S222D (Thermo Fisher) and 0.3-µM γP^32^ ATP (Perkin Elmer, Rodgau, Germany) at 37 °C in 80-mM HEPES pH 7.5, 4-mM MgCl_2_, 4-mM MnCl_2_, 1.6-mM DTT, and 70-µg/ml PEG_20 000_ for 15, 30, 45, and 60 min. The reaction was competed with 1000 × cold ATP or inhibited with 1.4-µM AZD6244. Heat-inactivated samples were separated with 10% polyacrylamide gels. The gels were dried and developed with imaging plates (BAS-IP MS 2340, Fujifilm, Japan).

### GO term analysis of MACC1 interactors

MACC1 interacting proteins were analyzed for GO term enrichment using DAVID v6.8 and GeneSCF v1.1, using standard parameters for background normalization. Visualization of GO term connections was performed by AmiGO.

### Structure prediction, structure representation, and electrostatic charge distribution

MACC1 protein sequence was employed for structure prediction PHYRE2. To visualize the predicted protein fold of MACC1, we used the Visual Molecular Dynamics (VMD) Molecular Graphics Viewer Version 1.9.3 package. MACC1 tyrosine phosphorylation sites were predicted using PROSCAN.

The electrostatic charge distribution on the surface of the predicted protein structure was calculated with PME electrostatics extension of VMD. VMD extension Mutate Residue was used for the in silico mutation of tyrosine to phenylalanine.

### Patients and tissues

For this study, 60 tumor and 4 normal tissue samples were used [4]. All patients gave their written consent (approved by Charité Ethics Committee, Charité-Universitätsmedizin, Berlin, Campus Mitte). The experiments conformed to the principles set out in the WMA Declaration of Helsinki and the Department of Health and Human Services Belmont Report. All patients were without familiar history of colon cancer, but were diagnosed with stage I–III colon cancer and did not receive any prior cancer treatment. Tumor specimens were collected during R0 resection and snap frozen.

### Anchorage-dependent cell proliferation

2 × 10^3^ per 96 well were seeded for 24 h. HGF was applied in serum-free medium. Cell viability was assessed by MTT assay following manufacturer’s instructions (Sigma Aldrich). MTT measurements were performed daily for 5 consecutive days in biological duplicates and technical triplicates.

### Wound healing assay

2.5 × 10^5^ or 1 × 10^5^ (drug treatment) cells were seeded to form a near-confluent monolayer before inflicting a wound. Wound closure was monitored daily for 4 days microscopically (Leica DM IL light microscope, Leica Microsystems, Leica Microsystems, Wetzlar, Germany) or 3 days (drug treatment) every second hour using the IncuCyte System (Essen Bioscience, Hertfordshire, UK). Directly after wound application cells were treated with indicated drug concentrations and combinations thereof. Image analysis was performed using the IncuCyte Scratch Wound Cell Migration Software Module licensed in the Zoom2016B Software (Essen Bioscience). Synergy was analyzed using combenefit 2.02. The wound healing experiments were performed three independent times, each in replicates.

### Colony formation

5 × 10^4^ cells were seeded in 0.8% soft agar/RPMI 10% FBS for 21 days. Colonies larger than four cells were counted. The colony-formation assay was performed two independent times in triplicate, and counted in quadruplicate.

### Scatter assay

2 × 10^3^ cells were seeded in 96-well plates for 24 h. Microphotographs were taken after 48 h following HGF application (20 U/ml). The scatter assay was performed two independent times in duplicate.

### Statistical analysis

Statistical analyses were performed with GraphPad Prism (Version 8, GraphPad Software, San Diego, CA). Levels of statistical significance for comparison of two groups were evaluated by using the non-parametric two-sided Mann–Whitney Test. To compare several groups with a control group, one-way ANOVA followed by Dunnett’s multiple comparison test was applied. To compare several groups with a control group over time, two-way ANOVA followed by Bonferroni posttest was applied. *P* values < 0.05 were considered to be statistically significant. Survival curves were generated using the Kaplan–Meier procedure. Survival curves and hazard ratios were evaluated using log-rank tests.

## Supplementary information

Supplementary Figure S1

Supplementary Figure S2

Supplementary Figure S3

Supplementary Movie S1

Supplementary Movie S2

Supplementary Figures and Tables

Supplementary Materials and Methods
