# Supplementary figures and images for: The newly identified MEK1 tyrosine phosphorylation target MACC1 is druggable by approved MEK1 inhibitors to restrict colorectal cancer metastasis
Source: Oncogene. 2021 Jul 10;40(34):5286–301. doi: 10.1038/s41388-021-01917-z (PMC8390371; doi:10.1038/s41388-021-01917-z)

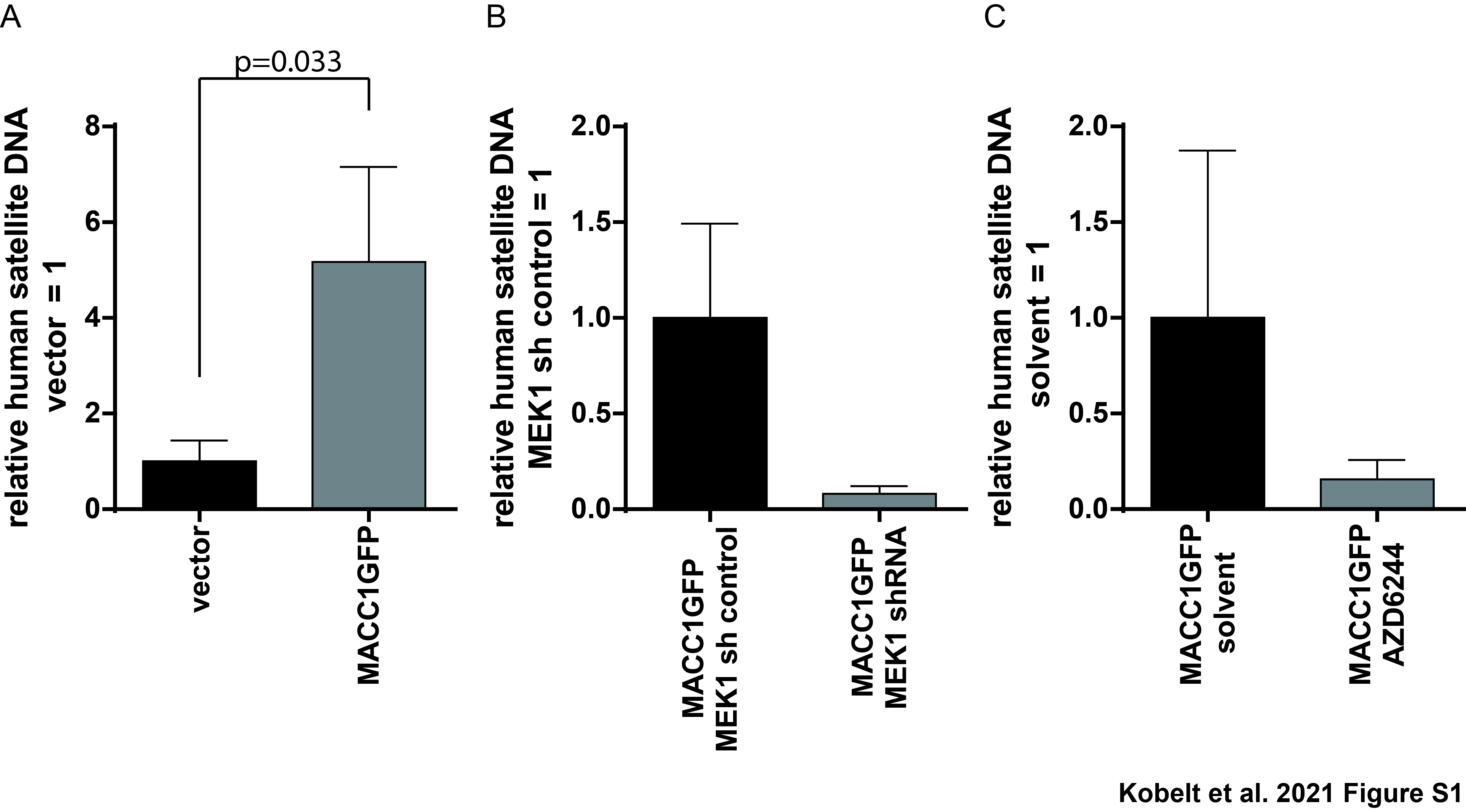

Supplement: Supplementary file 1 — Supplementary Figure S1 [file 41388_2021_1917_MOESM1_ESM.jpg]

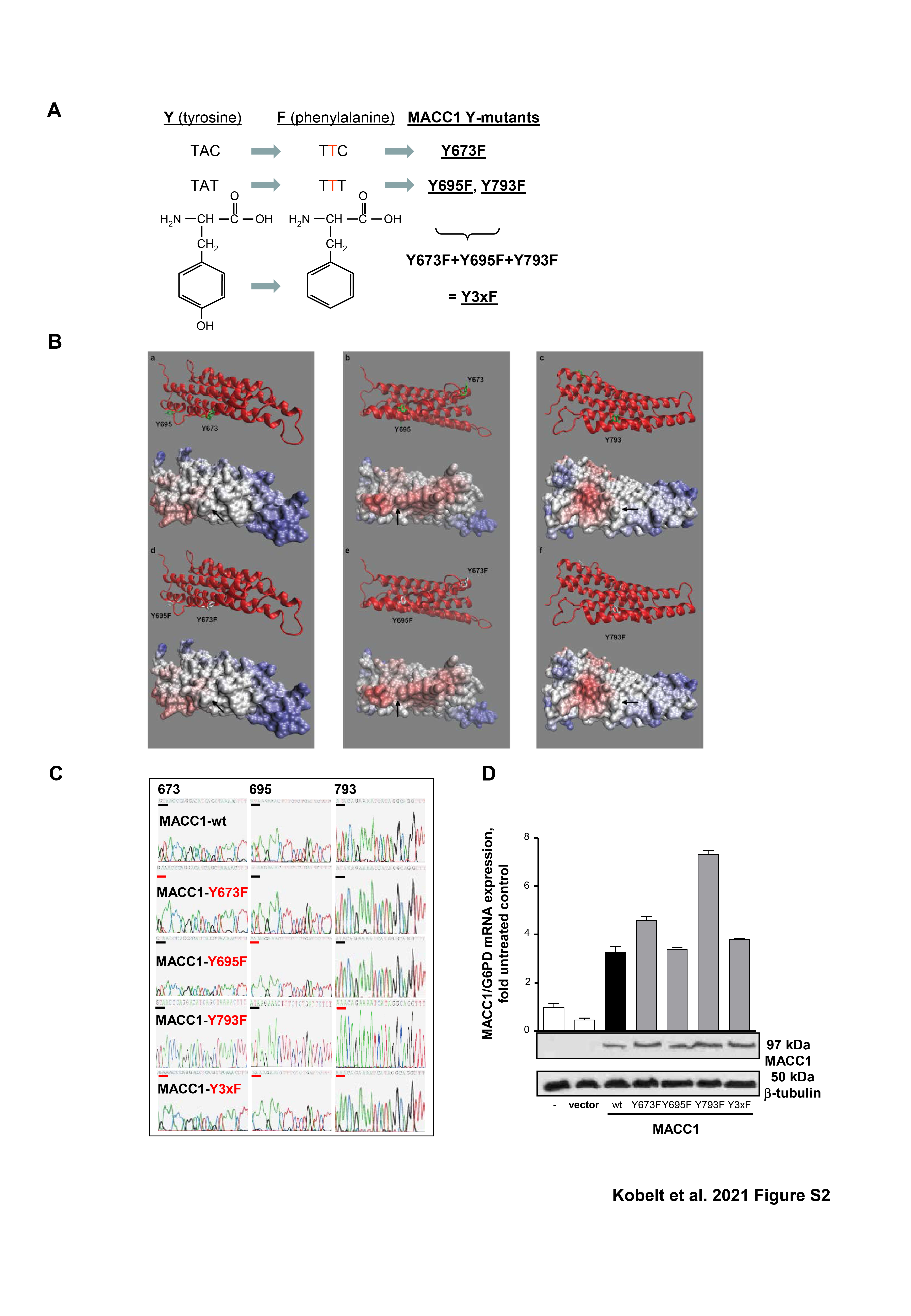

Supplement: Supplementary file 2 — Supplementary Figure S2 [file 41388_2021_1917_MOESM2_ESM.jpg]

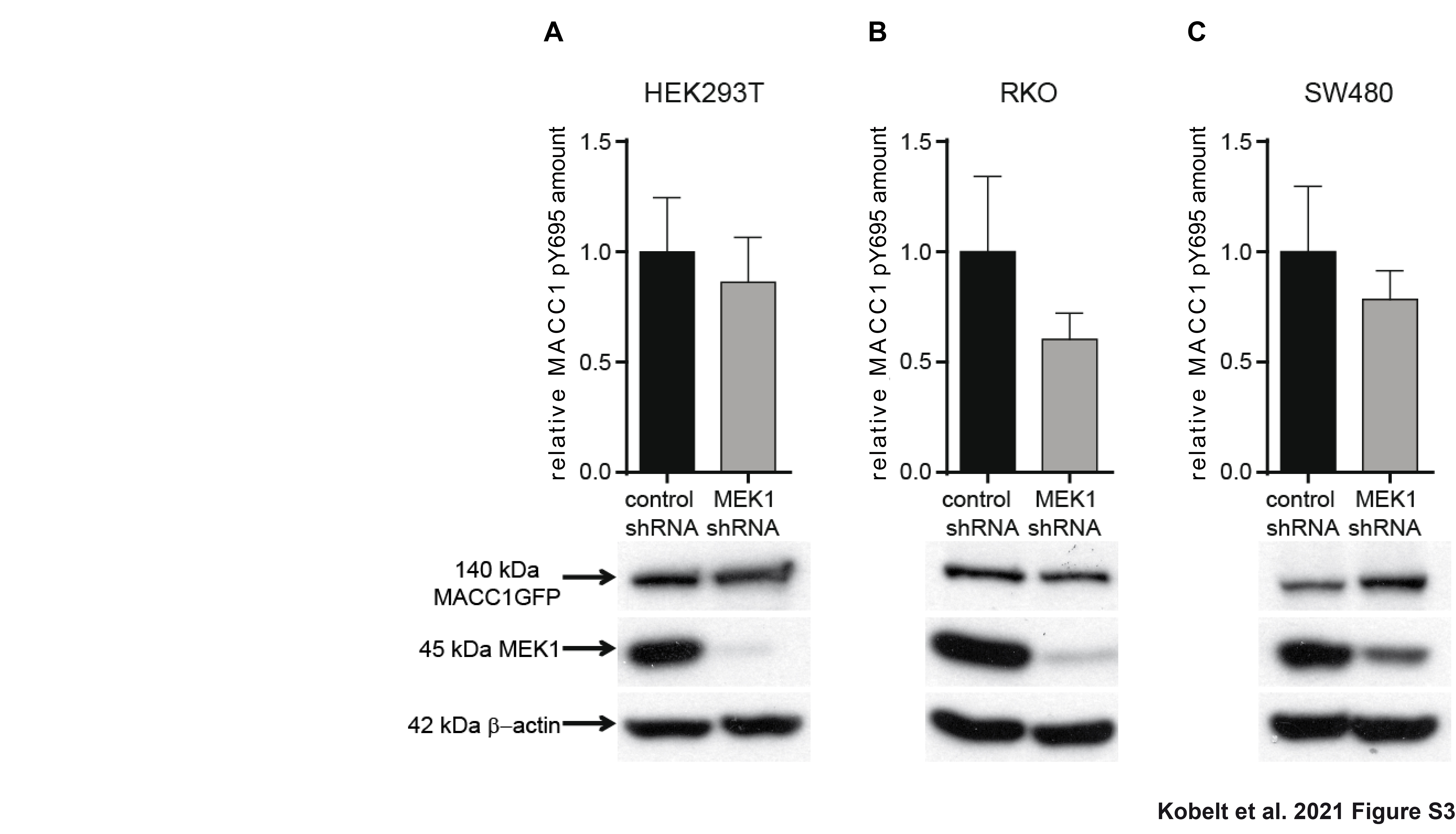

Supplement: Supplementary file 3 — Supplementary Figure S3 [file 41388_2021_1917_MOESM3_ESM.jpg]
