# Supplementary material for: The newly identified MEK1 tyrosine phosphorylation target MACC1 is druggable by approved MEK1 inhibitors to restrict colorectal cancer metastasis
Source: Oncogene. 2021 Jul 10;40(34):5286–301. doi: 10.1038/s41388-021-01917-z (PMC8390371; doi:10.1038/s41388-021-01917-z)
Supplement: Supplementary file 6 — Supplementary Figures and Tables [file 41388_2021_1917_MOESM6_ESM.docx]

**Supplementary figures and tables**

**Overview of supplementary figures**

**Figure S1.**

MACC1 overexpression and MEK1 inhibition in RKO cells in vivo.

**Figure S2.**

Site-directed mutagenesis of MACC1 tyrosine phosphorylation sites.

**Figure S3.**

Impact of MEK1 knockdown on phosphorylation of MACC1 in different cell lines.

**Overview of supplementary tables**

**Supplementary Table S1**

Model rank server output

**Supplementary Table S2**

Site-directed mutagenesis of tyrosine phosphorylation sites of MACC1.

**Supplementary Table S3**

Primers used for generation of MACC1 tyrosine phosphorylation mutants by site-directed mutagenesis.

**Supplementary Table S4**

Primers and probes used for qPCR of MACC1, Met, human satellite DNA (hus), MEK1 and G6PD.

**Supplementary Table S5**

A SRM-transitions used for the quantification of phospho-tyrosine peptides (QTRAP 4000)

B SRM-transitions used for the quantification of phospho-tyrosine peptide VSpYVIK (QTRAP 6500)

**Supplementary figures**

**
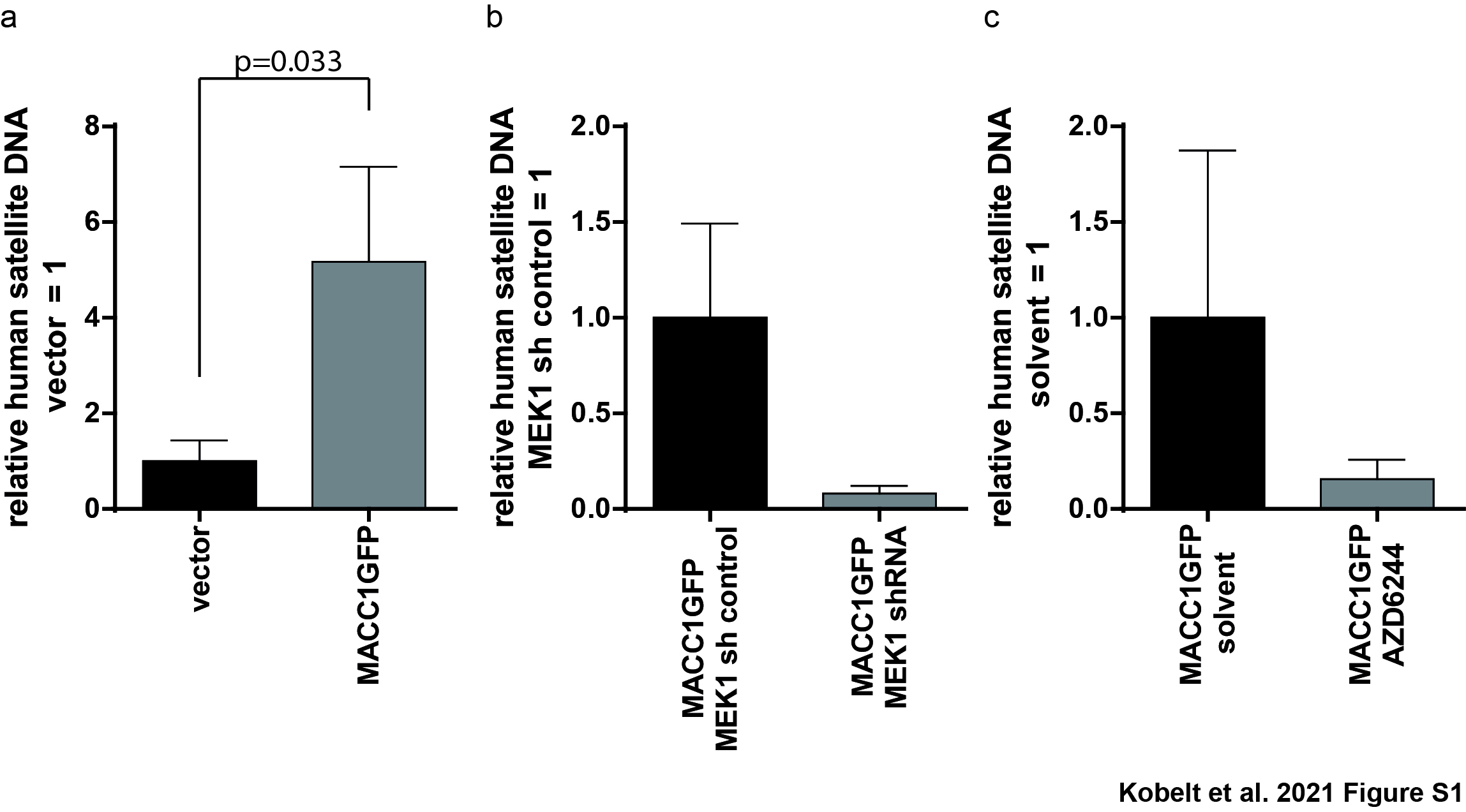
**

**Fig** **S1:** **MACC1 overexpression and MEK1 inhibition in RKO cells in vivo.**

Impact of MACC1 overexpression, MEK1 knockdown and treatment with MEK1 inhibitor AZD6244 on metastasis formation in mice measured as relative amount of human satellite DNA in mouse livers. Mice were intrasplenically transplanted with RKO/GFP, RKO/MACC1GFP, RKO/MACC1GFP sh-control, and RKO/MACC1GFP sh-MEK1 cells. Amount of human satellite DNA served as indicator of increased metastasis.

**a** RKO/vector and RKO/MACC1 cells were injected into the spleen of SCID beige mice. MACC1 overexpression increased the amount of metastasis as shown by significantly increased human satellite DNA (p=0.033) amount in livers of these animals. Data are presented as mean with SEM.

**b** RKO cells overexpressing MACC1 along with control or MEK1 shRNA were injected into the spleen of SCID beige mice. The amount of human satellite DNA was reduced upon reduction of MEK1. Data are presented as mean with SEM.

**c** RKO/vector and RKO/MACC1 cells were injected into the spleen of SCID beige mice. Animals were treated twice daily with solvent or the MEK1 inhibitor AZD6244 (50 mg/kg). The MEK1 inhibitor AZD6244 restricts MACC1-induced metastasis in mice. Data are presented as mean with SEM.

**
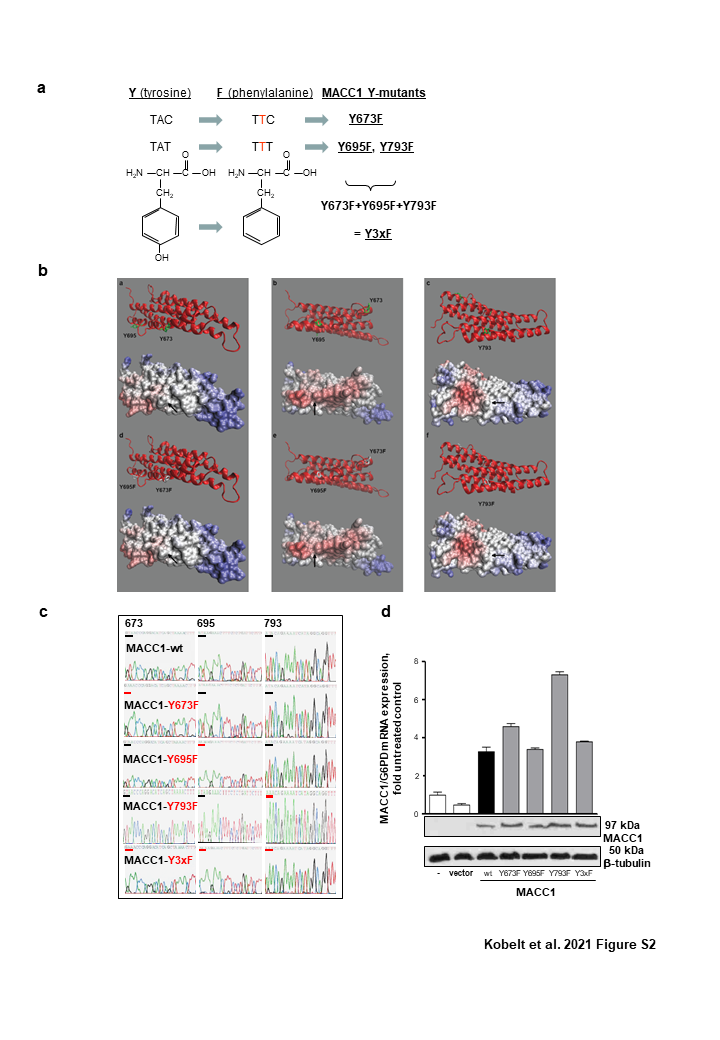
**

**Fig. S2: Site-directed mutagenesis of MACC1 tyrosine phosphorylation sites.**

**a** Site-directed mutagenesis of the potential tyrosine phosphorylation sites predicted by the PROSCAN algorithm at positions Y673, Y695 and Y793 of MACC1 to phenylalanines Y673F, Y695F, and Y793F. In addition a triple mutant harboring all 3 mutations was generated, Y3xF.

**b** Mutation of tyrosines to phenylalanines does not affect the local electrostatic charge distribution. a-c. Backbone view and space filling representation with electrostatic charge distribution of Y673, Y695, and Y793. Arrows indicate the critical oxygen atom of the tyrosine residues. d-f. Backbone view and space filling representation with electrostatic charge distribution after changing the tyrosines on the indicated positions to phenylalanines. The arrows now point to the missing oxygen atom.

**c** The electropherogram of the sequencing demonstrates confirmation of the site-directed mutagenesis of the constructs pcDNA3.1D/MACC1-wt, pcDNA3.1D/MACC1-Y673F, pcDNA3.1D/MACC1-Y695F, pcDNA3.1D/MACC1-Y793F, and pcDNA3.1D/MACC1-Y3xF (reverse complementary).

**d** Cell clones stably overexpressing the respective MACC1 pY-mutants were generated based on the cell line SW480, demonstrating virtually no endogenous MACC1 expression: SW480/MACC1-Y673F, SW480/MACC1-Y695F, SW480/MACC1-793F, and SW480/MACC1-Y3xF (in addition to SW480/vector and SW480/MACC1-wt; one representative clone is shown). Ectopic overexpression was determined by qRT-PCR and Western blotting.

**
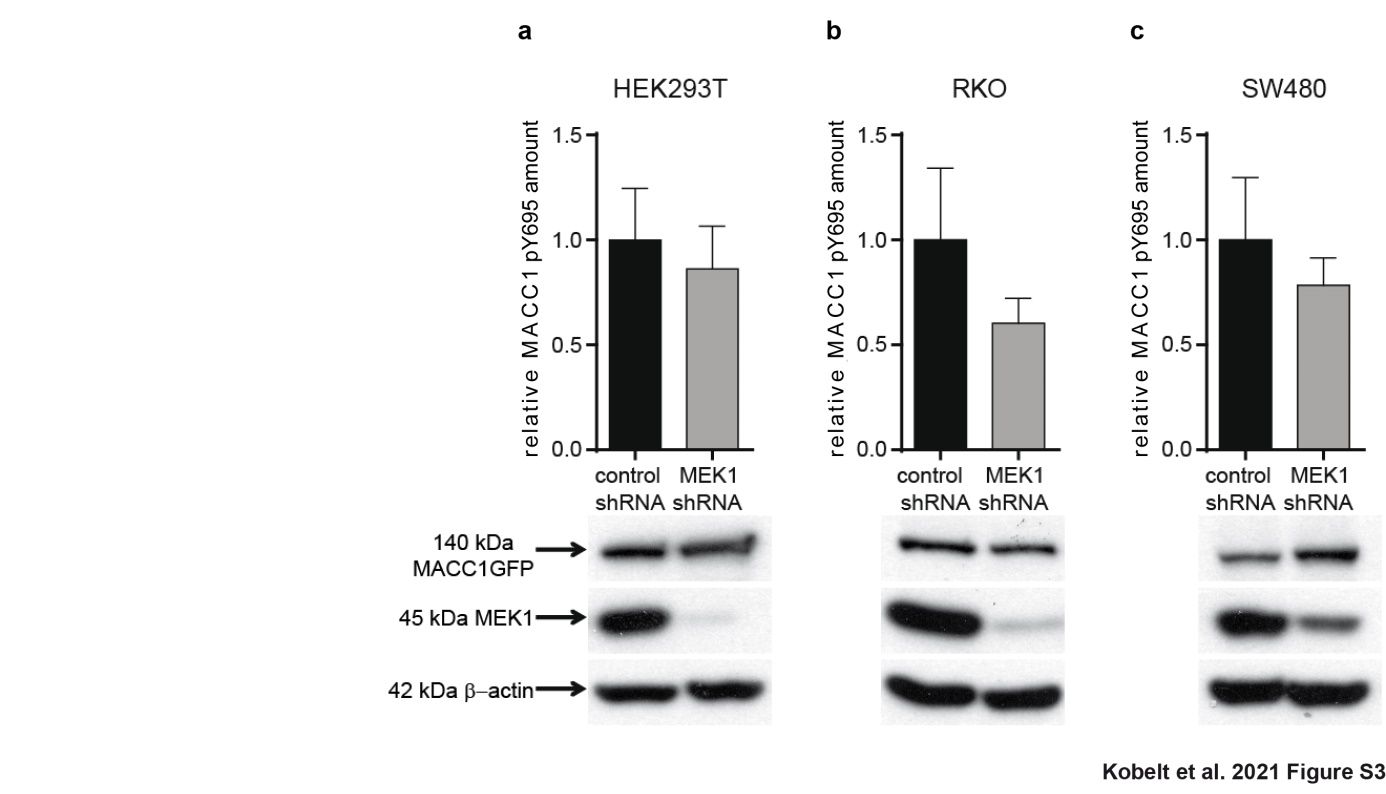
**

**Fig S3: Impact of MEK1 knockdown on phosphorylation of MACC1 in different cell lines.**

**a-c** Stable knock down of MEK1 decreases phosphorylation of MACC1 in **(a)** HEK293T cells, **(b)** RKO colon carcinoma cells and **(c)** SW480 colon carcinoma cells (peptide VSpYVIK). Mass spectrometry was performed 3 independent times, with 3 technical replicates in each experiment. Given are the relative amounts of MACC1 peptide VSpYVIK compared to control cells, expressed as mean with SEM. Western Blot analysis confirms MEK1 downregulation.

**Supplementary tables**

**Table S1**

**Model rank server output**

| **Rank** | **P-value** | **Score** | **uGDT/GDT** | **uSeqID/SeqID** | **ModelName** | **Template(s)** |
| --- | --- | --- | --- | --- | --- | --- |
| 1 | 3.6e-03 | 95 | 79/41 | 16/9 | 1t72A-49226_1 | 1t72A |
| 2 | 3.6e-03 | 95 | 84/40 | 33/16 | 2qnaA-49226_1 | 2qnaA |
| 3 | 3.9e-03 | 94 | 76/38 | 23/11 | 3gjxA-49226_1 | 3gjxA |
| 4 | 3.9e-03 | 94 | 75/36 | 17/8 | 1z3hA-49226_1 | 1z3hA |
| 5 | 3.0e-03 | 97 | 68/34 | 13/7 | 3s4wB-49226_1 | 3s4wB |

1) The model and alignment files under pdbs/ and alignments/ are named after $ModelName.pdb and $ModelName.fasta, respectively.

2) Please see http://raptorx.uchicago.edu/documentation/#goto2 for the explanation of P-value, Score, uGDT(GDT) and uSeqID(SeqID).

**Table S2**

**Site-directed mutagenesis of tyrosine phosphorylation sites of MACC1.**

| **MACC1 wt** | **Tyrosine Phosphorylation Site** | **SDM** | **MACC1 mutant** |
| --- | --- | --- | --- |
| **Y673** |  |  |  |
| nt | AAA GTT TTA GCT GAT GTC CTG GGT **TAC** | TTC | **Y673F** |
| aa 665 - 673 | K V L A D V L G **Y** | F |  |
| **Y695** |  |  |  |
| nt | AAA GAA TCA GAG AAA GTT TCT **TAT** | TTT | **Y695F** |
| aa 688 - 695 | K E S E K V S **Y** | F |  |
| **Y793** |  |  |  |
| nt | AAA CCT GCC TAT GAT TTT CTG **TAT** | TTT | **Y793F** |
| aa 786 - 793 | K P A Y D F L **Y** | F |  |

Nucleotides for introducing the mutations are underlined.

**Table S3**

**Primers used for generation of MACC1 tyrosine phosphorylation mutants by site-directed mutagenesis.**

| **T site** | **Primer** | |
| --- | --- | --- |
| **Y673F** | s | 5’-CTGATGTCCTGGGTT**T**CTCACATCTGTCCCTG-3’ |
|  | as | 5’-CAGGGACAGATGTGAG**A**AACCCAGGACATCAG-3’; |
| **Y695F** | s | 5’-GACAAAGAATCAGAGAAAGTTTCTT**T**TGTTATAAAGAAGTTAAAGGAAGATTGC-3’ |
|  | as | 5’-GCAATCTTCCTTTAACTTCTTTATAACA**A**AAGAAACTTTCTCTGATTCTTTGTC-3’ |
| **Y793F** | s | 5’-CTGCCTATGATTTTCTGT**T**TACCTGGAGTGCTCACTAT-3’ |
|  | as | 5’-ATAGTGAGCACTCCAGGTA**A**ACAGAAAATCATAGGCAG-3’ |

Nucleotides for introducing the mutations are underlined. s, sense; as, antisense

**Table S4**

**Primers and probes used for qPCR of MACC1, Met, human satellite DNA (hus), MEK1 and G6PD.**

| **Gene** | **Primer/Probe** | |
| --- | --- | --- |
| **MACC1** | forward | 5’-TTCTTTTGATTCCTCCGGTGA-3’ |
|  | reverse | 5’-ACTCTGATGGGCATGTGCTG-3’ |
|  | FITC probe | 5’-GCAGACTTCCTCAAGAAATTCTGGAAGATCTA-3’ |
|  | LCRed640 probe | 5’-AGTGTTTCAGAACTTCTGGACATTTTAGACGA-3’ |
| **Met** | forward | 5’-ATACGGTCCTATGGCTGGTG-3’ |
|  | reverse | 5’-TTTAACAGCAAACTCAGTTGAAATG-3’, |
|  | FITC probe | 5’-GAAAAACATGTACTTTAAAAAGTGTGTCAAACAG-3’ |
|  | LCRed640 probe | 5’-ATTCTTGAATGTTATACCCCAGCCCAAA-3’ |
| **hus** | forward | 5’- GGGATAATTTCAGCTGACTAAACAG-3’ |
|  | reverse | 5’-AAACGTCCACTTGCAGATTCTAG-3’ |
|  | FITC probe | 5’-CTTCACATAAAAACTACACAGATGCATTCTCAGG-3’ |
|  | LCRed640 probe | 5’-CTTTTTGGTGATGTTTGTATTCAACTCCCAG-3’ |
| **MEK1** | forward | 5’-CTGGTAGAGATGGCGGTTGG-3’ |
|  | reverse | 5’-CTCCACTGGGCAGTTTTGGA-3’ |
| **G6PD** | forward | 5’- ATCGACCACTACCTGGGCAA -3’ |
|  | reverse | 5’-CTCCACTGGGCAGTTTTGGA-3’ |

**Table S5**

**A SRM-transitions used for the quantification of phospho-tyrosine peptides (QTRAP 4000)**

| **Q1** | **Q3** | **Time** | **ID** | **DP** | **CE** |
| --- | --- | --- | --- | --- | --- |
| 1329,1 | 1692,8 | 250 | pY793 | 201 | 63 |
| 1329,1 | 1937,9 | 250 | pY793 | 236 | 63 |
| 1329,1 | 1407,7 | 250 | pY793 | 236 | 63 |
| 1329,1 | 1280,2 | 250 | pY793 | 231 | 63 |
| 544,8 | 601,3 | 250 | pY695 | 151 | 31 |
| 544,8 | 659,4 | 250 | pY695 | 156 | 31 |
| 544,8 | 688,3 | 250 | pY695 | 146 | 31 |
| 544,8 | 726,3 | 250 | pY695 | 141 | 31 |

Q1, mass selection on quadrupole 1; Q3, mass selection on quadrupole 3; Time, used for transition; ID, name of the transition; DP, declustering potential; CE, collision energy

**B SRM-transitions used for the quantification of phospho-tyrosine peptide VSpYVIK (QTRAP 6500)**

| **Q1** | **Q3** | **Time** | **ID** | **DP** | **CE** |
| --- | --- | --- | --- | --- | --- |
| 394.71 | 689.35 | 300 | 695 | 130 | 22.33 |
| 394.71 | 602.32 | 300 | 695 | 130 | 22.33 |
| 394.71 | 430.17 | 300 | 695 | 130 | 22.33 |
| 398.71 | 697.36 | 50 | 695 | 130 | 22.58 |
| 398.71 | 610.33 | 50 | 695 | 130 | 22.58 |
| 398.71 | 430.17 | 50 | 695 | 130 | 22.58 |

Q1, mass selection on quadrupole 1; Q3, mass selection on quadrupole 3; Time, used for transition; ID, name of the transition; DP, declustering potential; CE, collision energy
