# Supplementary material for: The newly identified MEK1 tyrosine phosphorylation target MACC1 is druggable by approved MEK1 inhibitors to restrict colorectal cancer metastasis
Source: Oncogene. 2021 Jul 10;40(34):5286–301. doi: 10.1038/s41388-021-01917-z (PMC8390371; doi:10.1038/s41388-021-01917-z)
Supplement: Supplementary file 7 — Supplementary Materials and Methods [file 41388_2021_1917_MOESM7_ESM.docx]

**Supplementary Materials and Methods**

**Drugs and treatments**

MEK1 inhibitors UO126 and PD98059 were obtained from Cell Signaling Technology (Danvers, MA, USA), AZD6244 and GSK1120212 from Selleck Chemicals (Munich, Germany) and were solubilized in DMSO. For migration experiments, cells were treated with 20 µM UO126, 50 µM PD98059, 10 µM AZD6244, and 10 µM GSK1120212, respectively, for 24 hours. DMSO-treated cells served as controls.

For in vivo application, 50 mg/kg AZD6244 was administered twice daily orally and 2 mg/kg GSK1120212 was given once daily orally (10% Kolliphor EL, Sigma Aldrich, Munich, Germany; 0.9% NaCl solution. Control mice were treated with the appropriate volume of 10% Kolliphor EL, 0.9% NaCl.

Recombinant HGF (kind gift of Walter Birchmeier, Berlin) was applied after serum starvation. Cells were harvested by scraping on ice after growth factor application. HGF activity was tested using MDCKC cells. One unit is defined as the amount HGF inducing scattering in this cell line.

**Plasmids and site-directed mutagenesis**

MACC1 full-length cDNA (+1 to +2556) was cloned in pcDNA3.1D/V5-His-Topo (Life Technologies, Carlsbad, CA, USA) vector (pcDNA3.1D/MACC1) (4). Point mutations were introduced by SDM (QuickChange Kit, Agilent, Santa Clara, CA, USA). The MACC1 full-length construct was used as template. Point mutations in the putative tyrosine (Y) phosphorylation sites Y673, Y695, and Y793 were inserted using primers (Supplementary Table S2 and S3, Biotez, Berlin, Germany) with the mutated sequence, resulting in phenylalanine (F) codons in MACC1: pcDNA3.1D/MACC1-Y673F, pcDNA3.1D/MACC1-Y695F, and pcDNA3.1D/MACC1-Y793F. A triple pY-mutated construct was generated sequentially starting with Y673F as a template, then introducing Y695F and Y793F (pcDNA3.1D/MACC1-Y3xF; Supplemental Fig. S2A). Generated plasmids were sequenced to confirm the mutations (Stratec Molecular, Berlin, Germany; Supplemental Fig. S2C).

**Cell lines and gene transfer**

Human cell lines SW480, SW620, RKO, HCT116, and HEK293T (American Type Culture Collection, Manassas, VA, USA) were maintained at 37^0^C in a humidified 5% carbon dioxide incubator in RPMI-1640 medium (SW480, HCT116) or DMEM (SW620, RKO, HEK293T) (Thermo Fisher, Waltham, MA) supplemented with 10% fetal bovine serum (FBS; Bio&Sell, Feucht, Germany). Cell lines were free of mycoplasma (MycoAlert Mycoplasma Detection Kit, Lonza, Basel, Switzerland). Authentication was performed by short tandem repeat (STR) genotyping at the DSMZ (German Collection of Microorganisms and Cell Cultures; Braunschweig, Germany). STR genotypes were consistent with published genotypes for these cell lines.

To generate SW480/vector, SW480/MACC1-wt, SW480/MACC1-Y673F, SW480/MACC1-Y695F, SW480/MACC1-Y793F and SW480/MACC1-Y3xF cells, the vector, MACC1-wt or pY-mutant MACC1 plasmids were stably transfected in SW480 cells (Lipofectin Transfection Reagent, Life Technologies). Clones were selected with 1.5 µg/ml Geneticin. MACC1 expression was analyzed by quantitative real-time RT-PCR (qRT-PCR) and Western blotting. For each experiment, at least 3 independently transfected clones were analyzed; one representative clone thereof is shown.

For testing the ability of MEK1 inhibitors to restrict MACC1-induced cell motility and MACC1-induced metastasis in mice by bioluminescence, we employed SW480 cells stably transfected with a bicistronic IRES vector (Clontech Laboratories, Mountain View, CA, USA) harboring luciferase and MACC1 (pIRES/luc-MACC1), resulting in SW480/luc-MACC1 cells.

MACC1-GFP, MEK1 shRNA and constitutively active MEK1 were stably introduced using lentiviruses at a MOI of 5. MEK1 (S218D, S222D)-pcw107 was a gift from David Sabatini and Kris Wood (Addgene plasmid #64604, Cambridge, MA, USA), pcw107 was a gift from John Doench and David Sabatini (Addgene, plasmid #62511). MACC1-GFP containing plasmid was purchased from Origene (Rockville, MD, USA), the control plasmid was generated by in-frame removal of MACC1. MEK1 shRNA containing plasmids were obtained from BioCat (Heidelberg, Germany).

**In vitro migration assay**

To investigate the impact of mutated MACC1 tyrosine phosphorylation sites on cell migration, 2.5 x 10^5^ SW480, SW480/vector, SW480/MACC1-wt, SW480/MACC1-Y673F, SW480/MACC1-Y695F, SW480/MACC1-Y793F, and SW480/MACC1-Y3xF cells were seeded into 12 mm diameter-transwell upper chamber with filter membranes (12 μm pore size, Merck, Darmstadt, Germany). RPMI-medium supplemented with 10% FBS was added to the bottom chamber. Cells were incubated for 24 hours. Cells that had migrated through the membrane into the lower chamber were counted in a Neubauer chamber (Roth, Karlsruhe, Germany). Cell migration assay was performed 3 times, each in duplicate. Each well was counted 6 times.

To investigate the impact of MEK1 inhibitors on MACC1-induced migration, SW480/luc-MACC1 cells were seeded in a 6 well plate and incubated overnight. Cells were treated with either DMSO, 20 µM UO126, 50 µM PD98059, 10 µM AZD6244, or 10 µM GSK1120212, for 2 hours. 2.5 x 10^5^ DMSO- or drug-treated cells were seeded into each transwell. Fresh medium supplemented with 10% FBS was added to the bottom chamber. Both chambers were treated with drugs or the respective volume of DMSO (37°C, 5% CO_2,_ 24 hours). Insets were removed and cells that migrated through the membrane to the lower chamber were counted as described above. The migration experiment was performed 3 times, each in triplicate. Each well was counted 4 times.

**Restriction of MACC1-induced metastasis in mice**

Experiments were performed in accordance with the United Kingdom Co-ordinated Committee on Cancer Research (UKCCCR) guidelines and approved by the responsible local authorities (State Office of Health and Social Affairs, Berlin, Germany).

For evaluating the impact of tyrosine phosphorylation for MACC1-induced metastasis in vivo, 6-week-old female non obese diabetic-severe combined immunodeficiency (NOD-SCID) animals were randomly assigned to 2 groups. 3 x 10^6^ SW480/MACC1-wt cells (n=16 mice) or SW480/MACC1-Y3xF cells (n=16 mice) were intrasplenically transplanted. To assess metastasis formation of MACC1-Y695F 3 x 10^6^ SW480/MACC1-wt cells (n=10 mice) or SW480/MACC1-Y695F cells (n=10 mice) were used. Animals were sacrificed by cervical dislocation at the experimental endpoint when the tumor reached the maximum size or when mice appeared moribund. Metastasis was evaluated by numbering and by scoring based on the calculation for each liver as the sum of the volumes of the individual metastases (length x width^2^ in mm^3^). To screen for human cells in mouse livers, organs were removed from all animals at the experimental endpoint, genomic DNA was isolated and human satellite DNA was analyzed by qPCR.

For evaluating the impact of MEK1 inhibitors on MACC1-induced metastasis in vivo, 6-week-old female SCID beige mice (Charles River, Sulzfeld, Germany) were randomly assigned in 3 groups. All mice were intrasplenically transplanted with 3 x 10^6^ SW480/luc-MACC1 cells.

Mice in the control group (n=6 mice) were treated daily with 2 ml/kg solvent orally (10% Kolliphor EL, 0.9% NaCl). Mice in the second group (n=6 mice) were treated with 50 mg/kg AZD6244 twice a day orally. Mice in the third group (n=6 mice) were treated with 2 mg/kg GSK1120212 once a day orally. Treatments started at the day of transplantation and were continued until the animals were sacrificed. Bioluminescence imaging was started 6 days after transplantation (NightOwl LB 981 systems, Berthold Technologies, Bad Wildbad, Germany), and performed twice per week. Animal experiments were terminated due to ethical reasons on day 39 by cervical dislocation in accordance with the local authorities. For bioluminescence imaging mice were anesthetized with isofluran (Abbott GmbH, Wiesbaden, Germany) and received intraperitoneally 150 mg/kg D-luciferin (Biosynth, Staad, Switzerland) dissolved in sterile PBS. Tumor growth and metastasis was monitored by WinLight (Berthold Technologies) and ImageJ 1.48k (NIH, Bethesda, MD) (82).

For quantification of metastasis, livers were removed and ex vivo bioluminescence imaging was performed at day 39. ImageJ version 1.48k was used for color coding of signal intensity (presenting a 256 grayscale) and overlay pictures. For detection of human cells in mouse livers at the experimental endpoint, genomic DNA was isolated from the livers of all animals and human satellite DNA was amplified by qPCR.

Similarly, for the RKO-based xenograft mouse model SCID beige mice were used. 3 x 10^6^ cells (RKO/vector, RKO/MACC1-GFP; RKO/MACC1-GFP/control shRNA, RKO/MACC1-GFP/MEK1 shRNA; RKO/MACC1-GFP for solvent and AZD6244 treatment) were transplanted intrasplenically. Anaesthetized mice were sacrificed by cervical dislocation. Human satellite DNA was analyzed by qPCR.

**RNA and qRT-PCR, DNA and qPCR**

RNA was isolated (TRIzol, Life Technologies). Two-step qRT-PCR was performed in parallel and in duplicate per sample (4). Briefly, 50 ng total RNA was reversely transcribed (random hexamer primers, 10 mM MgCl_2_, 1 × PCR-buffer II, 250 µM pooled dNTPs, 1 U/µL RNAse inhibitor, 2.5 U/µL MuLV reverse transcriptase; Life Technologies) at 42°C for 15 minutes, 95°C for 5 min, and cooling at 4°C for 5 min. Quantitative PCR was performed for 10 min at 95°C and 45 cycles of 10 sec at 95°C, 30 sec at 60°C, 4 sec at 72°C (LightCycler system, Roche). Calibrator cDNA for the genes of interest was derived from target cells and employed in serial dilutions simultaneously in each run. Mean values were calculated from duplicate qRT-PCR reactions, normalized to the housekeeping gene.

For detection of metastasized human cells in mouse livers following intrasplenic transplantation of SW480/MACC1 cells and treatment with the inhibitors AZD6244 and GSK1120212, genomic DNA was isolated from mouse livers (DNA-RNA-Protein Extraction Kit, Roboklon, Berlin, Germany). Quantitative PCR was performed using 50 ng genomic DNA. Titration was performed with genomic DNA from spiked human/mouse cell populations. Primer sequences (BioTeZ and TIB MolBiol, Berlin, Germany) are summarized in Supplementary Table S4.

**SRM-based quantification of the phosho-tyrosine containing peptides and shot-gun proteomics**

For quantification of tyrosine phosphorylation by SRM-MS, immunoprecipitated protein samples were solubilized in Laemmli loading buffer and subjected to SDS-PAGE. The gel band corresponding to the size of MACC1 was processed using an automated HTS PAL system (CTC Analytics, Switzerland) (81, 83). Peptides were extracted, purified and stored on reversed-phase (C_18_) StageTips following a protocol by Rappsilber et al. (84). After elution, the peptides were lyophilized and resuspended in (A) 0.1% formic acid / (B) 5% acetonitrile. Heavy-labeled (Lys 8) internal standard peptides (SpikeTides, JPT Inc., Berlin, Germany) were added at a level of 25 fmol/µg of protein. Each injection comprised of 1 μg of total protein. Peptides were separated on a 20 cm reversed-phase column, 75 μm ID, (C_18_ Reprosil, 3 μm, Dr. Maisch GmbH, Ammerbuch, Germany) using a gradient from 5% to 40% B in 90 min, and detected by a Q-Trap 4000 and 6500 (AB Sciex, Darmstadt, Germany).

SRM-methods were established by determining relevant transitions for the heavy (Lys 8) analogue. The interface heater temperature was 150°C, curtain gas was 30 psi, and ion spray voltage 2400 V. Applied collision gas was set to “high”. Q1 and Q3 were operated at unit resolution. The declustering potential (DP) was 130 V. The entrance potential (EP) and collision exit potential (CXP) were set as default. For the collision energy (CE) and time see Supplemental Table S5. All measurements were performed in positive mode. SRM-signals were quantified using the MultiQuant 3.0 software package (AB Sciex), the R-statistical software package and the ggplot2 module (85, 86). For SRM-transitions used for quantification of the phospho-tyrosine peptides see Supplemental Table S5. Three technical replicates of two independent biological replicates were measured and analyzed.

For identification of the MACC1 interactome by MS (shot-gun proteomics), IP of SW620 cells with two polyclonal rabbit anti-human MACC1 antibodies (HPA020103, HPA020081, Sigma, St. Louis, USA, 2 µg) was performed 4 times independently.

Samples were eluted from the affinity beads using denaturing buffer (6 M urea, 2 M thiourea, 20 mM HEPES, pH 8.0, Sigma). Proteins were converted to peptides in a two-step digestion using endopeptidase LysC (Wako, Japan) and trypsin (Promega, Madison, WI, USA). The peptides were desalted using Stage-Tips following a protocol by Rappsilber et al. (84). The purified peptides were then resuspended in 3% trifluor acetic acid / 5% acetonitrile buffer (Sigma, Merck) and separated on a reversed-phase column (20 cm length, 75 μm ID, 3 μm Reprosil-C18, Dr. Maisch) with a gradient from 5 to 45% acetonitrile in 122 min. Peptides were ionized on a Proxeon ion source and directly sprayed into the mass spectrometer (Q-Exactive, Thermo Fisher). The recorded spectra were analyzed using the MaxQuant software package (Version, 1.2.2.5) (87) with fixed modifications set to carbamylation of cysteines and variable modifications set to phosphorylation of serine, threonine, and tyrosine, and methionine oxidation. The false-discovery rate was set to 1% on protein and peptide level. Statistical analysis of the data set was performed using the R-statistical software package.

**Protein extraction, Western blotting and immunoprecipitation**

Cells were lysed in RIPA buffer (50 mM Tris pH 7.5; 0.15 M NaCl; 1% NP-40; 0.5% sodiumdeoxycholate) supplemented with complete protease inhibitor tablets (Roche), for 15 min on ice, harvested, and centrifuged (10 min, 14 000 rpm). Lysates of equal protein concentration were separated (NuPage 10% Bis-Tris gels, Life Technologies) and transferred to Hybond C Extra nitrocellulose membranes (GE Healthcare, Munich, Germany). Detection of V5-tagged MACC1 protein was performed with a direct HRP-labeled monoclonal mouse anti-human V5-specific antibody (Life Technologies, 1:2 500, overnight, 4°C), for MEK1 with a monoclonal mouse anti-human MEK1 antibody (Cell Signaling, 1:1 000, overnight, 4°C), and for β-tubulin with a monoclonal mouse anti-human β-tubulin antibody (BD, 1:1 000, overnight, 4°C). Antibody-protein-complexes were visualized with ECL reagent and exposure to CL-XPosure Films (Thermo Fisher Scientific).

To isolate GFP-tagged MACC1 the GFP Nanotrap kit (Chromotek, Planegg-Martinsried, Germany) was used. For cell lysis the supplied IP lysis buffer supplemented with cOmplete protease cocktail and phosSTOP phosphatase inhibitors (Roche) was used.

For the identification of the MACC1 interactome by MS, SW620 cells were employed for IP. 2 x 10^6^ cells were lysed with lysis buffer (20 mM Tris‑HCl pH 7.5, 150 mM NaCl), sonicated for 30 sec and centrifuged (10 min, 14 000 rpm, 4°C). A polyclonal rabbit anti-human MACC1 antibody was added to the supernatant (HPA020103, Sigma, 2 µg). As a technical replicate, a second polyclonal rabbit anti-human MACC1 antibody was used (HPA020081, Sigma, 2 µg). Incubations were performed overnight at 4°C. Both sets of experiments were performed in duplicate.

For detection of the physical binding of MACC1 and MEK1, 5 x 10^6^ SW480/MACC1-wt and SW620 cells were lysed (20 mM Tris-HCl pH 7.5, 150 mM NaCl, 0.1% NP40, 1 mM EDTA, 1% Triton-X-100, supplemented with protease inhibitor cocktail; 15 min on ice), harvested, and centrifuged (10 min, 14 000 rpm). The polyclonal rabbit anti-human MACC1 antibody (HPA020103, Sigma, 5 µg) was added (overnight, 4°C). IPs with an unrelated monoclonal mouse anti-human β-tubulin antibody (BD Bioscience, San Jose, CA, USA), 5 µg, overnight, 4°C,) served as negative control, IPs with a monoclonal mouse anti-human MEK1 antibody (Cell Signaling Technology, 5 µg, overnight, 4°C) served as positive control. One approach without IP served as technical control. 50 µl (50:50 v/v) Protein G Agarose beads (Life Technologies) were added (2 hours, 4°C). Beads were washed with lysis buffer. Elution was performed with 4 x SDS sample buffer (5 min, 95°C). Western blotting was carried out by using the monoclonal mouse anti-human MEK1 antibody (Cell Signaling Technology). To discriminate between MEK1 and MEK2 mouse monoclonal antibodies (H-8 and A-1, respectively) from Santa Cruz were used.

**Kinase assay**

10 µg MACC1-GFP from Nanotrap IP was incubated with 500 ng recombinant MEK1 with the activating mutations S218D S222D (Thermo Fisher) and 0.3 µM γP^32^ ATP (Perkin Elmer, Rodgau, Germany) at 37°C in 80 mM HEPES pH 7.5, 4 mM MgCl_2_, 4 mM MnCl_2_, 1.6 mM DTT and 70 µg/ml PEG_20 000_ for 15, 30, 45, and 60 min. The reaction was competed with 1 000 x cold ATP or inhibited with 1.4 µM AZD6244. Heat-inactivated samples were separated with 10% polyacrylamide gels. The gels were dried and developed with imaging plates (BAS-IP MS 2340, Fujifilm, Japan).

**GO term analysis of MACC1 interactors**

MACC1 interacting proteins were analyzed for GO term enrichment by the “Database for Annotation, Visualization and Integrated Discovery” (DAVID, v6.8) and “Gene Set Clustering based on Functional annotation” (GeneSCF, v1.1), using standard parameters for background normalization. Visualization of GO term connections was performed by AmiGO (88-90).

**Structure prediction, structure representation and electrostatic charge distribution**

MACC1 aa sequence (NCBI reference sequence NP_877439.3) was employed for structure prediction (Protein Homology/analogY Recognition Engine V 2.0 (PHYRE2), using the intense modelling mode (37). The detailed server output is available upon request. To visualize the predicted protein fold of MACC1, we used the Visual Molecular Dynamics (VMD) Molecular Graphics Viewer Version 1.9.3 package (91). MACC1 tyrosine phosphorylation sites were predicted using PROSCAN (36).

The electrostatic charge distribution on the surface of the predicted protein structure was calculated with PME electrostatics extension of VMD. VMD extension Mutate Residue was used for the in silico mutation of tyrosine to phenylalanine, followed by recalculation of electrostatic surface charge distribution with PME electrostatics.

**Patients and tissues**

For this study, 60 tumor and 4 normal tissue samples were used. All patients gave their written consent (approved by Charité Ethics Committee, Charité-Universitätsmedizin, Berlin, Campus Mitte). The experiments conformed to the principles set out in the WMA Declaration of Helsinki and the Department of Health and Human Services Belmont Report. All patients were without familiar history of colon cancer, but were diagnosed with stage I to III colon cancer and did not receive any prior cancer treatment. Tumor specimens were collected during R0 resection (complete resection with no microscopic residual tumor). Snap frozen samples were subjected to molecular analysis. For detailed patient characteristics and initial analysis of this cohort, see (4).

**Anchorage-dependent cell proliferation**

2 x 10^3^ SW480, SW480/vector, SW480/MACC1-wt, SW480/MACC1-Y673F, SW480/MACC1-Y695F, SW480/MACC1-Y793F, and SW480/MACC1-Y3xF cells/well were seeded in 96 well plates and incubated for 24 hours to allow the cells to attach to the bottom of the wells. Serum-free medium was added, supplemented with or without HGF. Viable cells were detected by addition of 3-(4,5-dimethyl-2-thiazol)-2,5-diphenyl-2H-tetrazolium bromide (MTT; Sigma-Aldrich) to a final concentration of 0.5 mg/ml (3 hours, 37°C). Reduced MTT was resolved using 10% SDS in 10 mM HCl and quantified at 560 nm in a 96 well plate reader. MTT measurements were performed daily for 5 consecutive days. The cell proliferation experiment was performed 2 independent times, each performed in triplicate.

**Wound healing assay**

2.5 x 10^5^ SW480, SW480/vector, SW480/MACC1-wt, SW480/MACC1-Y673F, SW480/MACC1-Y695F, SW480/MACC1-Y793F, and SW480/MACC1-Y3xF cells were grown to form sub-confluent monolayers. A wound of approximately 300 µm width was inflicted by cell scraping. Closure of the wound within the scrape line was inspected microscopically for 4 consecutive days (Leica DM IL light microscope, Leica Microsystems, Leica Microsystems, Wetzlar, Germany). The wound healing experiment was performed 3 independent times, each in duplicate.

For wound healing assays using the IncuCyte (Essen Bioscience, Hertfordshire, UK), 1x10^5^ cells were seeded in 96 well image lock plates (Essen Bioscience). Cells were allowed to adhere for 8 h. Wounds in the monolayer were applied using the IncuCyte Scratch Wound Cell Migration Kit (Essen Bioscience). Directly after wound application cells were treated with 1.25, 2.5 and 5 µM lovastatin and 0.01, 0.1 and 1 µM AZD6244 and all 9 combinations thereof. Cells treated with medium or 0.1% DMSO only served as controls. Pictures were taken every second hour for 72 h. Image analysis was performed using the IncuCyte Scratch Wound Cell Migration Software Module in conjunction with the IncuCyte ZOOM2016B software (Essen Bioscience). Synergy was analyzed using combenefit 2.02.

**Colony formation**

5 x 10^4^ SW480, SW480/vector, SW480/MACC1-wt, SW480/MACC1-Y673F, SW480/MACC1-Y695F, SW480/MACC1-Y793F, and SW480/MACC1-Y3xF cells were suspended in RPMI-medium supplemented with 10% FBS, mixed with 0.8% soft agar (Life Technologies), seeded in 6 cm culture dishes, and cultivated (37°C, 5% CO_2_) for 21 days. Only colonies consisting of at least 4 cells were considered. Colony formation was monitored microscopically (Leica DM IL light microscope). The colony formation assay was performed 2 independent times in triplicate, and counted in quadruplicate.

**Scatter assay**

2 x 10^3^ SW480, SW480/vector, SW480/MACC1-wt, SW480/MACC1-Y673F, SW480/MACC1-Y695F, SW480/MACC1-Y793F, and SW480/MACC1-Y3xF cells were seeded in 96 well plates. After 24 hours HGF (20 U/ml) was added. Cell phenotypes were documented 48 hours following HGF application. Scatter microphotographs were taken with a Leica DM IL light microscope (Leica Microsystems). The scatter assay was performed 2 independent times, each in duplicate.
